# Human 8-cell embryos enable efficient induction of disease-preventive mutations without off-target effect by cytosine base editor

**DOI:** 10.1093/procel/pwac043

**Published:** 2022-11-03

**Authors:** Yinghui Wei, Meiling Zhang, Jing Hu, Yingsi Zhou, Mingxing Xue, Jianhang Yin, Yuanhua Liu, Hu Feng, Ling Zhou, Zhifang Li, Dongshuang Wang, Zhiguo Zhang, Yin Zhou, Hongbin Liu, Ning Yao, Erwei Zuo, Jiazhi Hu, Yanzhi Du, Wen Li, Chunlong Xu, Hui Yang

**Affiliations:** Center for Reproductive Medicine, International Peace Maternity and Child Health Hospital, Innovative Research Team of High-level Local Universities in Shanghai, School of Medicine, Shanghai Jiao Tong University, Shanghai 200030, China; Institute of Neuroscience, State Key Laboratory of Neuroscience, Key Laboratory of Primate Neurobiology, CAS Center for Excellence in Brain Science and Intelligence Technology, Shanghai Institutes for Biological Sciences, Chinese Academy of Sciences, Shanghai 200031, China; Center for Reproductive Medicine, International Peace Maternity and Child Health Hospital, Innovative Research Team of High-level Local Universities in Shanghai, School of Medicine, Shanghai Jiao Tong University, Shanghai 200030, China; Center for Reproductive Medicine, Ren Ji Hospital, School of Medicine, Shanghai Key Laboratory for Assisted Reproduction and Reproductive Genetics, Shanghai Jiao Tong University, Shanghai 200127, China; Center for Reproductive Medicine, Anhui Provincial Maternal and Child Health Hospital, Hefei 230001, China; Institute of Neuroscience, State Key Laboratory of Neuroscience, Key Laboratory of Primate Neurobiology, CAS Center for Excellence in Brain Science and Intelligence Technology, Shanghai Institutes for Biological Sciences, Chinese Academy of Sciences, Shanghai 200031, China; Institute of Neuroscience, State Key Laboratory of Neuroscience, Key Laboratory of Primate Neurobiology, CAS Center for Excellence in Brain Science and Intelligence Technology, Shanghai Institutes for Biological Sciences, Chinese Academy of Sciences, Shanghai 200031, China; Institute of Neuroscience, State Key Laboratory of Neuroscience, Key Laboratory of Primate Neurobiology, CAS Center for Excellence in Brain Science and Intelligence Technology, Shanghai Institutes for Biological Sciences, Chinese Academy of Sciences, Shanghai 200031, China; The MOE Key Laboratory of Cell Proliferation and Differentiation, School of Life Sciences, Genome Editing Research Center, Peking University, Beijing 100871, China; Institute of Neuroscience, State Key Laboratory of Neuroscience, Key Laboratory of Primate Neurobiology, CAS Center for Excellence in Brain Science and Intelligence Technology, Shanghai Institutes for Biological Sciences, Chinese Academy of Sciences, Shanghai 200031, China; Shenzhen Branch, Guangdong Laboratory for Lingnan Modern Agriculture, Genome Analysis Laboratory of the Ministry of Agriculture, Agricultural Genomics Institute at Shenzhen, Chinese Academy of Agricultural Sciences, Shenzhen 518000, China; Shenzhen Branch, Guangdong Laboratory for Lingnan Modern Agriculture, Genome Analysis Laboratory of the Ministry of Agriculture, Agricultural Genomics Institute at Shenzhen, Chinese Academy of Agricultural Sciences, Shenzhen 518000, China; Shenzhen Branch, Guangdong Laboratory for Lingnan Modern Agriculture, Genome Analysis Laboratory of the Ministry of Agriculture, Agricultural Genomics Institute at Shenzhen, Chinese Academy of Agricultural Sciences, Shenzhen 518000, China; Center for Reproductive Medicine, Ren Ji Hospital, School of Medicine, Shanghai Key Laboratory for Assisted Reproduction and Reproductive Genetics, Shanghai Jiao Tong University, Shanghai 200127, China; Reproductive Medicine Center, Department of Obstetrics and Gynecology, the First Affiliated Hospital of Anhui Medical University, Hefei 230022, China; Center for Reproductive Medicine, Ren Ji Hospital, School of Medicine, Shanghai Key Laboratory for Assisted Reproduction and Reproductive Genetics, Shanghai Jiao Tong University, Shanghai 200127, China; Center for Reproductive Medicine, Shandong University, Jinan 250012, China; Center for Reproductive Medicine, Ren Ji Hospital, School of Medicine, Shanghai Key Laboratory for Assisted Reproduction and Reproductive Genetics, Shanghai Jiao Tong University, Shanghai 200127, China; Shenzhen Branch, Guangdong Laboratory for Lingnan Modern Agriculture, Genome Analysis Laboratory of the Ministry of Agriculture, Agricultural Genomics Institute at Shenzhen, Chinese Academy of Agricultural Sciences, Shenzhen 518000, China; The MOE Key Laboratory of Cell Proliferation and Differentiation, School of Life Sciences, Genome Editing Research Center, Peking University, Beijing 100871, China; Center for Reproductive Medicine, Ren Ji Hospital, School of Medicine, Shanghai Key Laboratory for Assisted Reproduction and Reproductive Genetics, Shanghai Jiao Tong University, Shanghai 200127, China; Center for Reproductive Medicine, International Peace Maternity and Child Health Hospital, Innovative Research Team of High-level Local Universities in Shanghai, School of Medicine, Shanghai Jiao Tong University, Shanghai 200030, China; Lingang Laboratory, Shanghai Research Center for Brain Science and Brain-Inspired Intelligence Technology, Shanghai 200031, China; Institute of Neuroscience, State Key Laboratory of Neuroscience, Key Laboratory of Primate Neurobiology, CAS Center for Excellence in Brain Science and Intelligence Technology, Shanghai Institutes for Biological Sciences, Chinese Academy of Sciences, Shanghai 200031, China

**Keywords:** human embryo, *APOE4*, disease-preventive mutations, base editor

## Abstract

Approximately 140 million people worldwide are homozygous carriers of *APOE4* (ε4), a strong genetic risk factor for late onset familial and sporadic Alzheimer’s disease (AD), 91% of whom will develop AD at earlier age than heterozygous carriers and noncarriers. Susceptibility to AD could be reduced by targeted editing of *APOE4*, but a technical basis for controlling the off-target effects of base editors is necessary to develop low-risk personalized gene therapies. Here, we first screened eight cytosine base editor variants at four injection stages (from 1- to 8-cell stage), and found that FNLS-YE1 variant in 8-cell embryos achieved the comparable base conversion rate (up to 100%) with the lowest bystander effects. In particular, 80% of AD-susceptible ε4 allele copies were converted to the AD-neutral ε3 allele in human ε4-carrying embryos. Stringent control measures combined with targeted deep sequencing, whole genome sequencing, and RNA sequencing showed no DNA or RNA off-target events in FNLS-YE1-treated human embryos or their derived stem cells. Furthermore, base editing with FNLS-YE1 showed no effects on embryo development to the blastocyst stage. Finally, we also demonstrated FNLS-YE1 could introduce known protective variants in human embryos to potentially reduce human susceptivity to systemic lupus erythematosus and familial hypercholesterolemia. Our study therefore suggests that base editing with FNLS-YE1 can efficiently and safely introduce known preventive variants in 8-cell human embryos, a potential approach for reducing human susceptibility to AD or other genetic diseases.

## Introduction

Alzheimer’s disease (AD) represents one of the most severe and widespread neurodegenerative diseases due to a progressive loss of memory and other cognitive functions (Huang et al., 2012; Selkoe, 1991). Moreover, with no effective treatments for AD currently available, the number of AD patients increases at an astounding doubling rate of 20 years, which if unchecked, will result in 80 million affected individuals by 2040. Mutations in the genes encoding amyloid precursor protein (*APP*), presenilin-1 (*PSEN1*), and presenilin-2 (*PSEN2*) are associated with early-onset familial AD, but only accounts for >1% of all AD cases (Huang and Mucke, 2012; Selkoe, 1991). The majority of AD cases occur at age 65 or older, and are thus commonly referred to as late-onset AD. In humans, the *APOE* gene has three major isoforms [*APOE2* (ε2), *APOE3* (ε3), and *APOE4* (ε4)] that each confer different effects on lipid and neuronal homeostasis (Tanzi, 2012).

Genetically, *APOE4* (~13% frequency in the general population) serves as the single greatest risk factor for late-onset familial and sporadic AD (Farrer et al., 1997; Reiman et al., 2020; Tanzi, 2012; Zlokovic, 2013), which together account for 65%–70% of AD cases (Farrer et al., 1997). The mean age and frequency of AD at clinical onset in ε4 homozygotes are 68 years and 91%, respectively, 76 years of age and 47% in ε4 heterozygotes, and 84 years and 20% in ε4 noncarriers (Corder et al., 1993; Liu et al., 2013b; Rebeck et al., 1993), indicating that *APOE4* leads to a dramatically increased risk of AD and lowers the age of disease onset in a gene-dose-dependent manner (Huang and Mucke, 2012). Compared with the most common allele, *APOE3,* the *APOE2* allele has been reported to provide protective effects (Reiman et al., 2020), whereas *APOE4* increases the risk of late-onset sporadic AD by 3-fold for heterozygous carriers and 15-fold for homozygous carriers (Raber et al., 2004; Smith et al., 2019a). Mechanistically, *APOE4* has been shown to cause late-onset neurodegeneration via amyloid-β (Aβ)-dependent or Aβ-independent pathways (Huang et al., 2014; Mahley et al., 2006), and recently also shown to induce tau neurofibrillary degeneration (Ossenkoppele et al., 2015; Serrano-Pozo et al., 2015; Wennberg et al., 2018), astrocyte and microglia responses (Alvin Huang et al., 2019; Iannucci et al., 2021; Lin et al., 2018), and blood–brain barrier disruption (Halliday et al., 2016; Montagne et al., 2020), which ultimately increase the risk of sporadic AD.

An estimated 140 million people carry homozygous copies of ε4 worldwide, rendering the possibility of using preimplantation genetic diagnosis to identify ε4 couples and eliminate homozygous ε4 embryos during conception unrealistic at best, even among the limited set of carriers undergoing assisted reproductive therapy. Indeed, only a single amino acid difference distinguishes the ε4 (112R/158R; CGC/CGC) allele from ε3 (112C/158R or 112R/158C; TGC/CGC or CGC/TGC), which could be efficiently converted from a C to T without inducing a double-strand break (DSB) in human cells (Komor et al., 2016) and embryos using a cytosine base editor (CBE) such as a Cas9 nickase with a fused deaminase. Since homozygous *APOE4* carriers represent a much larger population (i.e., millions of adults) than that of other mutation carriers with severe, premature death-associated monogenic defects, targeted editing of *APOE4* in embryos could provide broad therapeutic benefits for a massive swath of high-risk candidates of late-onset sporadic AD.

Conventional CRISPR/Cas9 triggers an error-prone, nonhomologous end joining DNA repair pathway after DSB induction, resulting in complex repairing alleles such as insertion, deletion, substitutions, and other compound mutations potentially with loss-of-function, gain-of-function, and other unknown consequences. Furthermore, a recent study found that CRISPR-Cas9 administration in early human embryos leads to frequent loss of the targeted chromosome and uncontrollable chromosome rearrangement (Zuccaro et al., 2020), raising huge safety concerns about this technology. BE using nickase Cas9 and engineered deaminase could introduce targeted base conversions without DSB, providing a much safer genome editing tool without causing detrimental off-target effects than wildtype Cas9 (Doman et al., 2020; Gaudelli et al., 2017; Komor et al., 2016; Zuo et al., 2020). Previous studies (Dang et al., 2022; Liang et al., 2017; Zeng et al., 2018; Zhang et al., 2019) have demonstrated induction and correction of genetic mutations with base editors (BE) in human embryos. However, whether base editing could install preventive mutations in human embryos with high efficiency and specificity remains to be investigated.

To test whether susceptibility to this widespread and untreatable disease could be reduced in high risk ε4-carrying embryos, we investigated the possibility of converting ε4 to ε3 alleles by targeted base editing while performing stringent control measures to confirm that only the correct edits were introduced. To this end, we first screened eight variants of CBEs by injection at four different embryonic stages (from the 1- to 8-cell stages) and found that the FNLS-YE1 editor in 8-cell embryos resulted in the comparable base conversion rate (i.e., up to 100%) with the lowest bystander [nontargeted nucleotide substitution (NTS) or indel mutation] effects. Further assays demonstrated that FNLS-YE1 could efficiently convert AD-susceptible ε4 allele into the AD-neutral ε3 allele in human embryos carriers for ε4. In addition, we also found that FNLS-YE1 could efficiently introduce protective alleles in *TYK2* (Diogo et al., 2015; Harper et al., 2015)/*WDFY4* (Yang et al., 2010; Zhang et al., 2014) and *PCSK9*/*ANGPTL4* (Harper et al., 2015; Stitziel et al., 2016), offering a potential approach to reduce susceptivity for systemic lupus erythematosus (SLE) and familial hypercholesterolemia (FH), respectively. Overall, these findings in 8-cell embryos provide a technical basis that can guide the development of low-risk personalized gene therapies *in utero*, and somatic editing approaches for reduced risk of AD or other genetic diseases.

## Results

### FNLS-YE1 shows high efficiency of cytosine base editing with the fewest bystander effects in 8-cell human embryos

**Figure 1.**
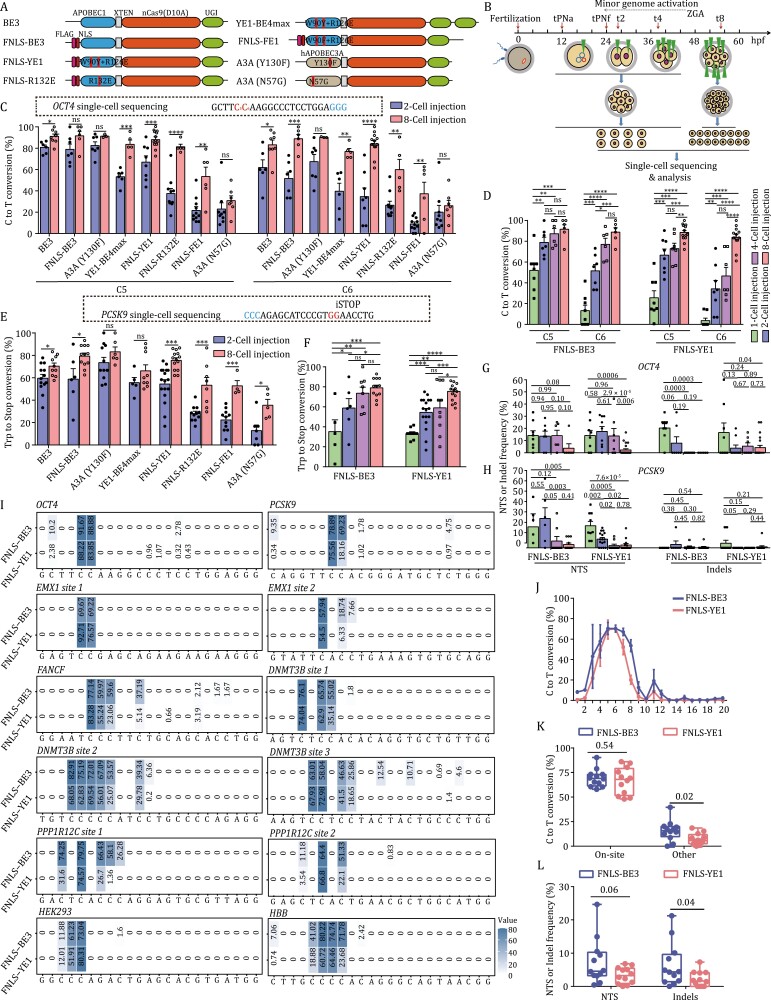
**Improved base-editing efficiency of different CBE variants in human 3PN cleavage embryos compared with zygotes.** (A) Schematic showing the architectures of eight CBEs used in this study. Linkers between functional domains are shown as yellow boxes. FNLS, Flag-tagged nuclear localization signal; UGI, uracil glycosylase inhibitor; YE1 = BE3^W90Y + R126E^; R132E = BE3^R132E^; FE1 = BE3^W90F + R126E^; (B) Experimental design for base editing efficiency assessment after CBE injection at different stages of cleaving embryos. Different reagent mixtures were injected into zygote, 2-cell, 4-cell, or 8-cell stage embryos. Embryos were cultured to the 8-cell or early morula stage for single-cell Sanger sequencing. ZGA, zygotic genome activation; tPNa, i.e., time when pronuclei appear, which reflects the beginning of the first embryonic interphase; tPNf, i.e., time when pronuclei faded and entry into the first embryonic M-phase. The t2, t4, and t8 parameters are defined as the times for achieving the stage characterized by the corresponding number of cells (t2 for 2 cells, t4 for 4 cells, and t8 for 8 cells). (C) Single-cell sequencing analysis of base editing efficiency in embryos injected with eight CBE variants targeting the *OCT4* locus at the 2-cell and 8-cell stages. Percentage of alleles with targeted C > T conversions on *OCT4* is shown. sgRNA and PAM sequences are shown in black and blue, respectively. Target nucleotides within the CBE editing window are shown in red. (D) Single-cell sequencing analysis of base editing efficiency in embryos injected with FNLS-BE3 and FNLS-YE1 targeting the *OCT4* locus at the 1-cell, 2-cell, 4-cell, and 8-cell stages. (E) Single-cell sequencing analysis of base editing efficiency in embryos injected with eight CBE variants targeting the *PCSK9* locus at the 2-cell and 8-cell stages. Percentage of alleles with targeted conversion of *Trp* codon to stop codon conversion on the *PCSK9* locus. iSTOP, induction of stop codon. (F) Single-cell sequencing analysis of base editing efficiency in embryos injected with FNLS-BE3 and FNLS-YE1 targeting the *PCSK9* locus at the 1-cell, 2-cell, 4-cell, and 8-cell stages. (G and H) Frequency of NTSs and indel mutations in human embryos injected with FNLS-BE3 and FNLS-YE1 targeting the *OCT4* (G) and *PCSK9* (H) loci at the 1-cell, 2-cell, 4-cell, and 8-cell stages. Indels, indel mutations; NTS, NTS. (I) Heatmaps showing C-to-T editing efficiency on 12 human endogenous sites at the 8-cell stage in embryos injected with FNLS-BE3 and FNLS-YE1. (J) Editing window analysis based on the C-to-T editing efficiency of FNLS-BE3 and FNLS-YE1 on the 12 target sites. (K) Comparison of FNLS-BE3 and FNLS-YE1 activity for on-site C-to-T and bystander editing efficiency on the 12 target sites. On-site, positions 5–7. Other, positions outside 5–7. The center line indicates the median, and the bottom and top lines of the box represent the first quartile and third quartile of the values, respectively. Tails extend to the minimum and maximum values. (L) The indels and NTS frequency on the 12 target sites edited with FNLS-BE3 and FNLS-YE1. *n* = 12 independent experiments for each group in (J–L). Data are presented as the mean ± SEM. *P < 0.05, **P < 0.01, ***P < 0.001, ****P < 0.0001, unpaired Student’s *t*-test. ns, not significant. Each dot in Fig. 1C–H and 1K–L represents an embryo and the endogenous locus tested, respectively.

In previous work, we showed that 2-cell human embryos could be robustly edited using a cytosine base editor 3 (BE3) (Zhang et al., 2019). To determine whether other BEs could function with similar effectiveness, we systematically compared eight BEs, including BE3 (Komor et al., 2016), FNLS-BE3 (Zuo et al., 2020), A3A (Y130F) (Wang et al., 2018), YE1-BE4max (Doman et al., 2020), FNLS-YE1 (Zuo et al., 2020), FNLS-R132E (Zuo et al., 2020), FNLS-FE1 (Zuo et al., 2020), and A3A (N57G) (Gehrke et al., 2018) in human embryos derived from discarded 3-pronucleus (PN) zygotes (Figs. 1A and S1A). We also investigated the dependence of base editing efficiency on cleavage stage by examining the activity of different BE variants in 1, 2, 4, and 8-cell embryos. To this end, we injected mRNAs encoding each BE together with a single-guide RNA (sgRNA) into blastomeres at each of these stages (Fig. 1B). Targeting the *OCT4* gene, we found that BE3, FNLS-BE3, A3A (Y130F), YE1-BE4max, FNLS-YE1, and FNLS-R132E induced higher C-to-T or Gln to Stop condon conversion rates than those induced by FNLS-FE1 and A3A (N57G) (Figs. 1C and S1C). Moreover, the editing efficiency in 8-cell embryos was significantly higher than that in 2-cell embryos for nearly all variants (Fig. 1C). In particular, FNLS-YE1 mediated 88.2% and 83.9% conversion at g.187C>T (C5) and g.188C>T (C6) in 8-cell embryos, respectively, as compared to 66.7% and 34.1% in 2-cell embryos (Fig. 1C). In comparison with FNLS-BE3, FNLS-YE1 was previously shown to exhibit very high fidelity in mouse embryos and human cell lines, inducing no detectable DNA or RNA off-target effects (Doman et al., 2020; Zuo et al., 2020). We thus selected FNLS-YE1 and FNLS-BE3 for further comprehensive evaluation of their C-to-T conversion rates in the *OCT4* and *PCSK9* genes of 1-, 2-, 4-, and 8-cell stage embryos (Fig. 1D–F) and found that both FNLS-BE3 and FNLS-YE1 exhibited the highest base editing efficiency in 8-cell embryos (Fig. 1D–F). Furthermore, we found that FNLS-YE1 also displayed highly efficient (>75%) STOP codon conversion (CAA to TAA and TGG to TGA/TAG/TAA) in the *OCT4* and *PCSK9* genes of 8-cell embryos (Figs. 1E, 1F, S1C and S1D).

Next, we checked for bystander effects resulting from either FNLS-BE3 or FNLS-YE1 base editing in the *OCT4* and *PCSK9* genes of different early-cleavage embryos. We found that both editors induced fewer NTS or indel mutations in 8-cell embryos than in 1/2/4-cell embryos (Figs. 1G, 1H and S1E–H). These findings suggesting the advantages of 8-cell embryos were further confirmed using a panel of 10 other endogenous genes (Fig. 1I). Further comparison of the two editors showed that FNLS-YE1 had a similar editing window and editing efficiency as FNLS-BE3, but with significantly fewer bystander effects (Figs. 1J–L, S1I and S1J). Finally, we verified that the editing effects we observed in 8-cell embryos were not due to the total amount of injected editing agents. For this purpose, we used a 4-fold increase in the concentration of editing agents in 2-cell embryos and found no significant changes in efficiency for FNLS-YE1 (Fig. S1B).

Taken together, our results identified FNLS-YE1 as the most efficient and accurate editor among all eight editors tested here, and that the 8-cell stage was the most suitable for base editing in early human embryos.

### FNLS-YE1 induces no detectable off-target effects or developmental defects

**Figure 2.**
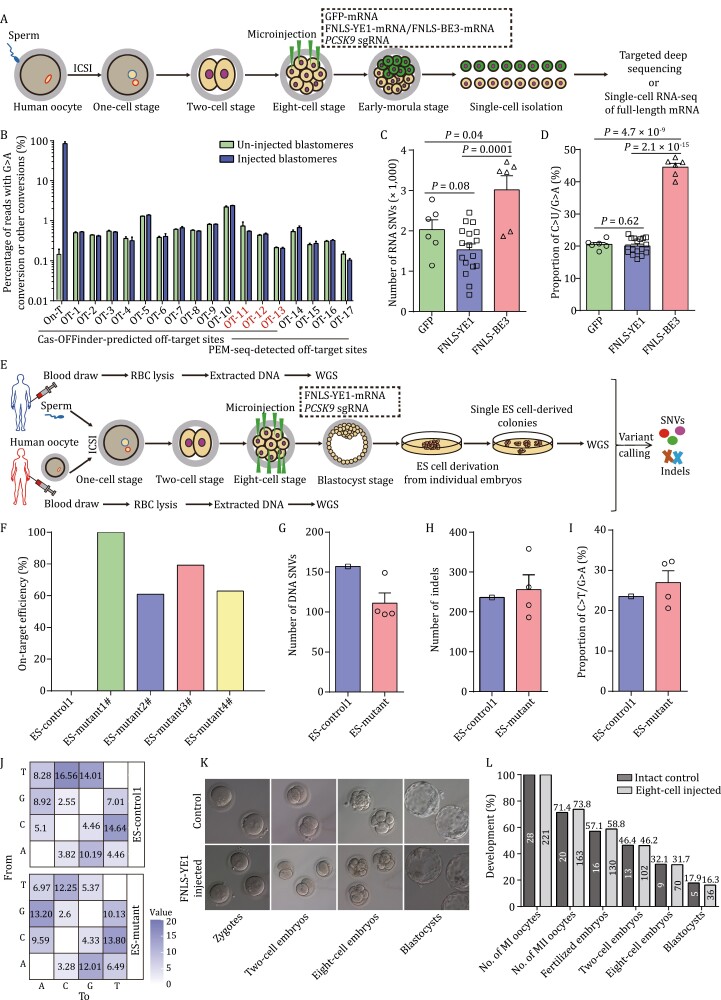
**Integrated evaluation of off-target and developmental effect of FNLS-YE1 in human 2PN embryos and derived stem cells.** (A) Schematic of sgRNA-dependent DNA off-targeting and sgRNA-independent RNA off-targeting analyses using targeted deep sequencing and RNA sequencing of FNLS-YE1-treated embryos undergoing whole-genome/whole-transcriptome amplification at the single cell level. FNLS-YE1 mRNA/FNLS-BE3 mRNA, *PCSK9* sgRNA, and GFP mRNA or only GFP mRNA were co-injected into four blastomeres of 8-cell embryos whereas the other four blastomeres were left un-injected. When embryos developed to the early morula stage, GFP-positive and negative blastomeres were separated and analyzed by targeted deep sequencing or RNA-seq. (B) Genome-wide analysis of sgRNA-dependent off-target events with PEM-seq and targeted deep sequencing in human blastomeres with or without base editing. A schematic diagram of the PEM-seq workflow is shown in Fig. S2A. (C) Number of off-target SNVs identified by RNA-seq in human blastomeres injected with FNLS-YE1/*PCSK9*/GFP or FNLS-BE3/*PCSK9*/GFP compared with those injected with GFP only. (D) Proportion of C·G to U·A mutations identified by RNA-seq for GFP, FNLS-YE1/*PCSK9*/GFP, and FNLS-BE3/*PCSK9*/GFP groups. (E) Schematic of sgRNA-independent off-target analysis with WGS of hESCs derived from FNLS-YE1-treated or untreated embryos. FNLS-YE1 mRNA and *PCSK9* sgRNA were co-injected into 8-cell stage embryos. Subsequently, hESCs were derived until injected embryos developed into blastocysts after trophectoderm biopsy and isolation of inner cell mass into a 4-well plate. Ultimately, genomic DNA of hESCs was extracted for WGS. In addition, peripheral blood genomes of both parents were used as a background control. (F) Percentage of on-target efficiency for one control and four independent hESCs derived from FNLS-YE1-treated and untreated embryos analyzed by WGS. (G and H) Number of SNVs (G) and indels (H) identified in one control and four independent hESCs by WGS. (I) Proportion of C·G to T·A mutations detected in hESCs derived from control and *PCSK9*-edited embryos using FNLS-YE1. (J) Distribution frequency of SNV mutation types. The number in each cell indicates the proportion of a certain type of mutation among all SNV mutations. (K) Representative morphology of the control embryos and the FNLS-YE1-injected embryos at different cleavage stages. (L) Developmental rate from MI-stage oocyte to blastocyst for un-injected and injected embryos with FNLS-YE1 targeting *PCSK9*. Data are presented as the mean ± SEM. *P* values were evaluated with unpaired Student’s *t*-test. Each dot in Fig. 2C–D and 2G–I represents a single blastomere and an embryonic stem cell line, respectively.

Recent studies have reported that CBEs can induce both Cas9-dependent (resulted from sequence similarity) (Fu et al., 2013; Hsu et al., 2013) and Cas9-independent (resulted from deaminase) off-target effects in both genomic DNA (Zuo et al., 2019) and transcriptome-wide mRNA (Grunewald et al., 2019b; Zhou et al., 2019). However, some engineered BEs, including FNLS-YE1, were shown to induce no detectable off-target effects in mouse embryos and human cell lines (Doman et al., 2020; Zuo et al., 2020). We therefore sought to further verify whether FNLS-YE1 induced off-target effects in human embryos. We first examined whether FNLS-YE1 induced any Cas9-dependent off-target alterations. To minimize the differences in genetic background between the gene-edited and control blastomeres, we co-injected FNLS-YE1, GFP mRNA, and *PCSK9* sgRNA for g.55046590G>A (G8) or g.55046591G>A (G7) into 4 of the 8 blastomeres of an eight-cell embryo derived from a 2PN zygote (Fig. 2A). The injected blastomeres were identified by GFP expression (GFP^+^) in early morula embryos. We found no evidence indicating the introduction of off-target mutation in targeted deep sequencing analysis of the 13 top off-target sites predicted by Cas-OFFinder in either the injected or un-injected blastomeres (Fig. 2B). Furthermore, we also conducted primer extension mediated (PEM)-seq experiments to identify potential off-target loci of the *PCSK9* sgRNA in HEK293T and human embryonic stem cell lines (hESCs). This analysis captured seven off-target mutations in the Cas9/sgRNA-treated cells (Fig. S2). Comparison between injected with un-injected blastomeres showed that none of these seven off-target loci had a significant editing signal in targeted deep sequencing data (Fig. 2B). These results thus indicated that FNLS-YE1 induced no Cas9-dependent off-target alterations apart from its targeted edits in *PCSK9* at g.55046590G>A (G8) or g.55046591G>A (G7) in 8-cell embryos.

To next test for Cas9-independent off-target effects on RNA, we performed single-cell RNA sequencing at two days after injecting blastomeres with either the *“*GFP-only,” “FNLS-YE1, *PCSK9* sgRNA, and GFP,” or “FNLS-BE3, *PCSK9* sgRNA, and GFP” RNAs, together with corresponding un-injected control blastomeres (Fig. 2A). We found no differences in the background levels of RNAs harboring single nucleotide varients (SNVs), nor in the pattern of canonical C-to-U conversions in the blastomeres injected with FNLS-YE1 compared with the GFP only-injected control blastomeres (Fig. 2C and 2D). By contrast, FNLS-BE3 induced severe off-target effects in the transcriptome and canonical C-to-U pattern in human blastomeres, which was consistent with previous studies (Figs. 2C, 2D, S3 and S4) (Grunewald et al., 2019a, 2019b; Zhou et al., 2019).

Third, we performed whole genome sequencing (WGS) on hESCs derived from 8-cell human embryos injected or not with FNLS-YE1, together with sgRNAs targeting *PCSK9*. We also sequenced and analyzed the genomes of peripheral blood cells obtained from sperm and egg donors as a control for genetic background to distinguish FNLS-YE1-induced off-target variants from germline variants (Fig. 2E). Targeted deep sequencing was used to confirm the high targeting efficiency of FNLS-YE1 in four hESCs derived from edited human embryos (Fig. 2F). WGS showed that the numbers of SNVs and indels were similar between FNLS-YE1-edited and nonedited hESCs (Figs. 2G, 2H, S5A and S5B). Furthermore, these SNVs for each edited hESCs exhibited no canonical patterns of C-to-T conversion characteristic of off-target activity by CBEs (Figs. 2I, 2J and S5C) (Zuo et al., 2019, 2020). We also performed karyotyping analysis of different hESCs and somatic cells collected from the gamete donors, and found normal karyotypes in all cells, indicating no obvious chromosomal abnormalities after base editing by FNLS-YE1 (Table S1). In addition, targeted deep sequencing analysis of the top 13 off-target sites predicted by Cas-OFFinder showed no off-target mutations in the FNLS-YE1-edited and nonedited hESCs, nor in the seven potential off-target sites identified by PEM-seq (Fig. S5D). Taken together, these PEM-seq, RNA sequencing, and WGS results demonstrated the high targeting specificity of FNLS-YE1 in human embryos without detectable off-target effects.

Finally, to investigate the potential effects of FNLS-YE1 editing on embryonic development, we examined the morphology and development rates of embryos from the 8-cell to blastocyst stages injected or not with FNLS-YE1. We observed no morphological abnormalities in the 8-cell injected embryos compared with that of un-injected control embryos (Fig. 2K). Developmental rates also appeared unaffected in the 8-cell injected embryos, implying that base-editing with FNLS-YE1 was safe for use in embryonic editing (Fig. 2L; Table S2).

### Conversion of AD-susceptible ε4 to the AD-neutral ε3 alleles in human 3PN embryos

**Figure 3.**
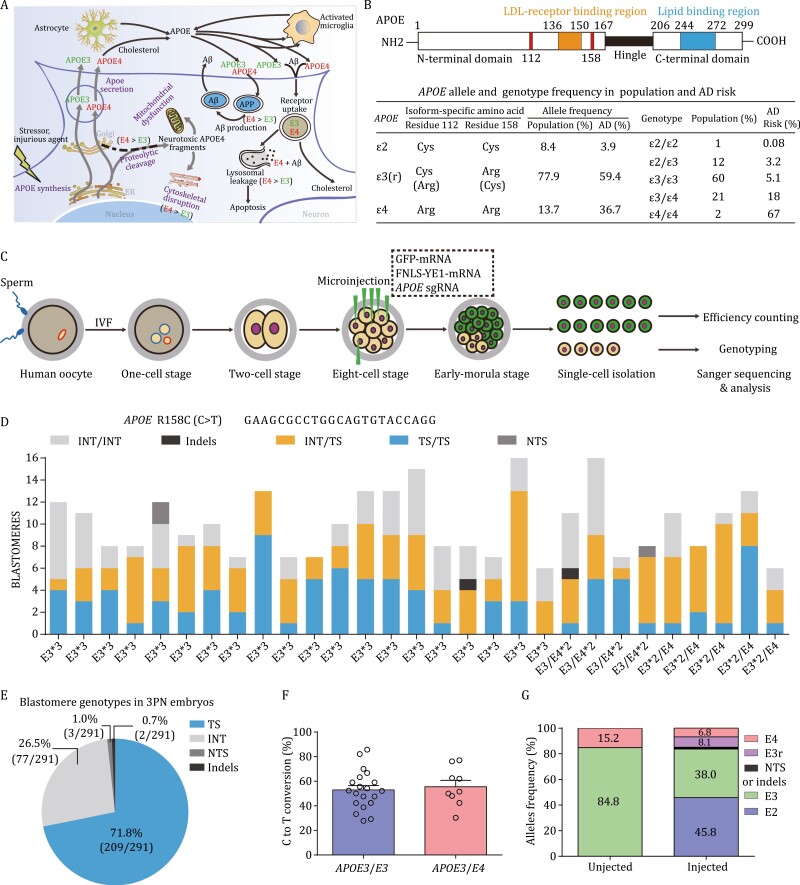
**Robust correction of Alzheimer’s disease associated *APOE4* allele in human 3PN embryos with high-fidelity FNLS-YE1.** (A) Roles of *APOE* in Aβ-dependent and Aβ-independent pathways in AD pathology; the detrimental effects of *APOE4* reflect its unique structural and biophysical properties. APOE is synthesized by astrocytes, activated microglia, and neurons. In response to stressors and injurious agents, neurons turn on or increase their expression of APOE to repair or remodel the damaged neurons. However, neuronal APOE undergoes proteolytic processing and APOE4 is more susceptible than APOE3 to the cleavage. The APOE fragments cause cytoskeletal disruption, such as tau phosphorylation, neurofibrillary tangle (NFT) formation, and mitochondrial dysfunction, finally leading to neurodegeneration. Figure adapted from (Mahley et al., 2006). (B) APOE structure and genotype frequency in a human population. Data from (Liu et al., 2013a; Raber et al., 2004). (C) Experimental diagram showing FNLS-YE1-mediated gene correction of the *APOE4* locus in human 3PN embryos. (D) Percentage of nonedited and edited blastomeres carrying targeted substitution (TS) or NTS/indel mutations in human 3PN embryos injected with FNLS-YE1 targeting *APOE4* or *APOE3* alleles (158R) at the 8-cell stage. (E) Blastomere genotyping results of embryos injected with FNLS-YE1 targeting the *APOE4* or *APOE3* loci in 3PN human embryos. (F) Percentage of alleles with targeted C to T conversion for FNLS-YE1 in human 3PN embryos carrying *APOE3/E3* and *APOE3/E4*. Each dot represents an embryo. (G) Summarized allelic frequency change before and after FNLS-YE1 injection.

The strongest genetic risk factor for late-onset AD, apolipoprotein E (*APOE*), appears to increase risk largely by influencing Aβ seeding and clearance (Fig. 3A) (Castellano et al., 2011; Verghese et al., 2013). Variants at amino acids 112 and 158 were classified as *APOE2* (ε2, 112C/158C), *APOE3* [ε3, 112C/158R; ε3r, 112R/158C (Seripa et al., 2011, 2007)], and *APOE4* (ε4, 112R/158R) (Fig. 3B), among which *APOE* ε2/ε3r have been shown or are presumed (Seripa et al., 2007, 2011) to confer lower risk of AD than *APOE4*. In principle, conversion of the high risk ε4 allele to the neutral ε3(r) and protective ε2 allele via one and two sequential C-to-T substitutions (at 112/158), respectively, could confer protective effects against AD development (Fig. 3B).

To investigate the efficacy of FNLS-YE1 C-to-T conversion in the *APOE* gene in human embryos, we co-injected GFP mRNA, FNLS-YE1 mRNA and *APOE*-targeting sgRNA for ε4 (g.44908822C>T, ε4>ε3r) and ε3 (g.44908822C>T, ε3>ε2) together into six blastomeres of 8-cell human embryos derived from 3PN zygotes (Fig. 3C). Genotype analysis of the two un-injected blastomeres showed that 20/29 3PN embryos carried the ε3 allele, while 9/29 carried both the ε3 and ε4 alleles (Fig. 3D). Genotype analysis of the FNLS-YE1-injected blastomeres showed that 209/291 (71.8%) of the injected blastomeres had C-to-T editing events, resulting in ε3-to-ε2 or ε4-to-ε3r conversion (Fig. 3E). Overall, this treatment resulted in 53.0% and 55.5% C-to-T conversion at the allelic level for ε3/ε3 and ε3/ε4 embryos, respectively (Fig. 3F). Specifically, C-to-T editing of the ε3 allele resulted in a marked increase in the frequency of the protective ε2 allele from 0% to 45.8% (Fig. 3G). Furthermore, greater than half of the ε4 allele copies were converted to the ε3r allele (Fig. 3G).

### Efficient conversion of *APOE* ε4 to ε3 allele in ε4-carrying 2PN human embryos

**Figure 4.**
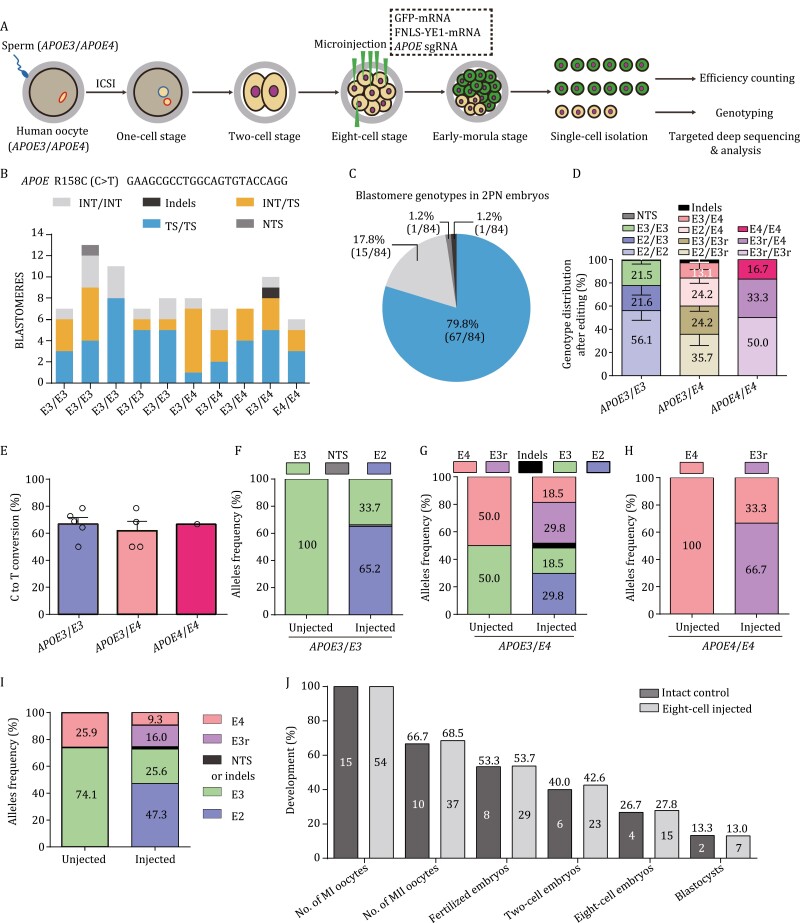
**Efficient conversion of AD risk variant ε4 to neutral variant ε3 in ε4 carrying 2PN embryos.** (A) Experimental diagram showing FNLS-YE1-mediated gene correction of the *APOE4* locus in human 2PN embryos. Sperm from a heterozygous male (*APOE4*/*APOE3*) was used to fertilize the oocytes to obtain 2PN embryos by ICSI. FNLS-YE1 mRNA, *APOE4*-targeting sgRNA, and GFP mRNA were co-injected into six blastomeres of 8-cell embryos whereas the other blastomeres were left un-injected. When embryos developed to the early morula stage, GFP-positive and negative blastomeres were separated and analyzed by targeted deep sequencing. (B and C) Blastomere genotyping results of embryos injected with FNLS-YE1 targeting the *APOE4* or *APOE3* loci (158R) in 2PN human embryos. (D) Genotype change induced by FNLS-YE1 in single blastomere from human 2PN embryos carrying *APOE3*/*E3*, *APOE3*/*E4*, and *APOE4*/*E4*. (E) Percentage of alleles with targeted C to T conversion for FNLS-YE1 in human 2PN embryos carrying *APOE3*/*E3*, *APOE3*/*E4*, and *APOE4*/*E4*. Each dot represents an embryo. (F–H) Allelic frequency changes induced by FNLS-YE1 in human embryos carrying *APOE3*/*E3* (F), *APOE3*/*E4* (G), and *APOE4*/*E4* (H) respectively. (I) Summarized allelic frequency change before and after FNLS-YE1 injection. (J) Developmental rate from MI-stage oocyte to blastocyst for un-injected and injected embryos with FNLS-YE1 targeting *APOE*. Data are presented as the mean ± SEM.

Based on the editing results with 3PN embryos, we performed similar experiments on 2PN embryos generated by intracytoplasmic injection with sperm derived from an ε3/ε4 donor (Fig. 4A). We then performed *APOE* targeting in 10 cleaving embryos, including five ε3/ε3, four ε3/ε4, and one ε4/ε4 carriers (Fig. 4B) and found that 67/84 (79.8%) of the analyzed blastomeres had C-to-T conversion of either the ε3 or ε4 alleles (Fig. 4B and 4C). For ε3/ε3 embryos, 26/46 (56.1%) blastomeres became ε2/ε2 and 10/46 (21.6%) became ε2/ε3 (Figs. 4D and S6A). Furthermore, 5/6 (80%) blastomeres of one ε4/ε4 embryo showed ε4>ε3r conversion in at least one ε4 allele, and 3/6 (50%) blastomeres became ε4-free by converting both ε4 alleles to ε3r alleles (Figs. 4D and S6A). Overall, >60% C-to-T conversion at the allelic level was achieved for 2PN embryos (Fig. 4E), indicating an increase from 0% to 47.3% in the protective ε2 allele, while over half of the ε4 alleles were converted to the ε3r alleles (Fig. 4F–I), similar to that observed in 3PN embryos. Furthermore, NTS and indel analysis showed that FNLS-YE1 editing of the *APOE* locus induced <2% of unwanted mutations (Fig. S6B). In addition, we also examined whether FNLS-YE1 could edit *APOE* position 112R to achieve ε4>ε3 (g.44908684C>T) conversion. Since we found no NGG PAM at the target site, PAMless-FNLS-YE1 (Walton et al., 2020) was used to induce C-to-T conversion. However, using three different sgRNAs, we still observed no conversion of ε4 to ε3 (Fig. S6C).

**Figure 5.**
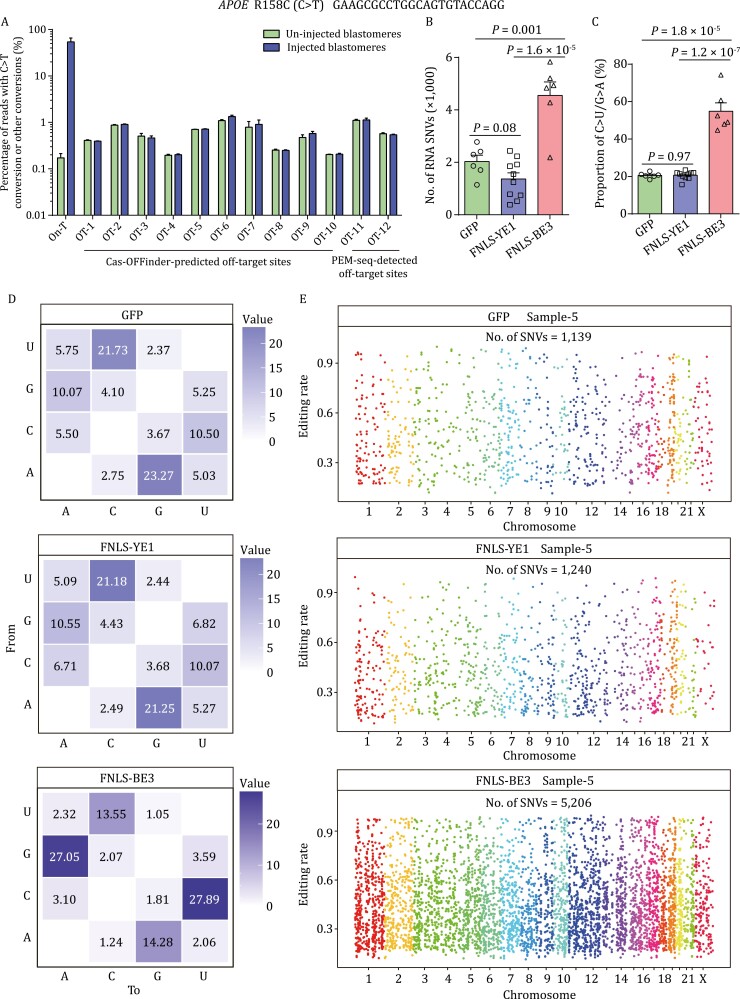
**Off-target evaluation of FNLS-YE1 targeting *APOE* (158R) in human 2PN embryos.** (A) Targeted deep sequencing analysis of the on-target and potential off-target loci predicted by Cas-OFFinder and captured by PEM-seq for *APOE* (158R) in human blastomeres with or without FNLS-YE1 injection. Data are presented as the mean ± SEM. (B) Number of off-target SNVs identified by RNA-seq in human blastomeres injected with FNLS-YE1/*APOE*/GFP or FNLS-BE3/*APOE*/GFP compared with those injected with GFP only. (C) Proportion of C·G to U·A mutations identified by RNA-seq for GFP, FNLS-YE1/*APOE*/GFP, and FNLS-BE3/*APOE*/GFP groups. (D) Transcriptomic SNVs distribution for different base conversion types detected in single blastomere of injected embryos for *APOE* (158R) editing. (E) Representative distributions of off-target RNA SNVs on human chromosomes for GFP, FNLS-YE1/*APOE*/GFP, and FNLS-BE3/*APOE*/GFP groups. Chromosomes are indicated with different colors. Data are presented as the mean ± SEM. *P* values were evaluated with unpaired Student’s *t*-test. Each dot in Fig. 5B and 5C represents a single blastomere.

Analysis of developmental rates identified no developmental defects in the edited embryos from the 8-cell to blastocyst stages (Fig. 4J). We also used targeted deep sequencing to check the off-target sites for *APOE* (R158C) and found no evidence of off-target editing (Figs. 5A and S7). Furthermore, we performed single blastomere RNA sequencing of *APOE*-edited embryos to screen for potential off-target effects in the transcriptome. In contrast to the conventional FNLS-BE3 editor, *APOE*-targeting by FNLS-YE1 resulted in similar numbers of SNVs and C-to-U patterns as the GFP group at the RNA level (Figs. 5B–E and S8), indicating the absence of detectable off-target effects, in agreement with the results of *PCSK9*-targeting experiments.

Taken together, these results suggest that it is feasible to reduce the risk of AD associated with the *APOE4* allele without inducing off-target effects using the high-fidelity FNLS-YE1 editor via sequential conversion of *APOE4* into the neutral *APOE3r* or *APOE3* into the protective *APOE2* alleles.

### Induction of various protective mutations for other diseases

**Figure 6.**
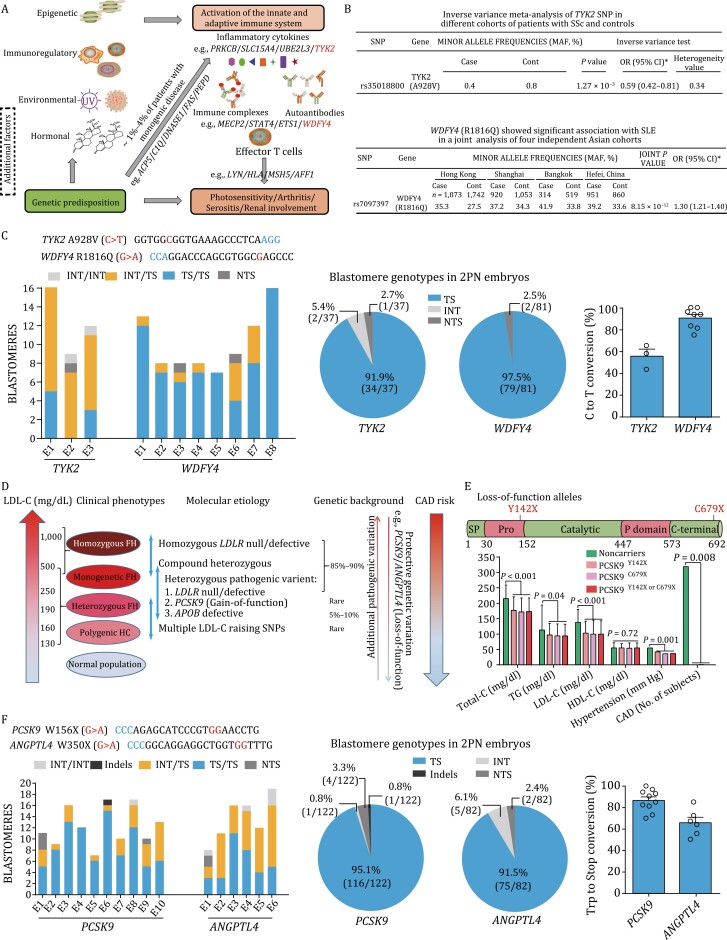
**Efficient induction of protective mutation for SLE and FH in human 2PN embryos with high-fidelity FNLS-YE1.** (A) Risk factors associated with SLE. Figure is adapted from (Smith et al., 2019b). (B) Protective alleles for SLE identified on *TYK2* and *WDFY4* genes by previous genome-wide association study (GWAS). Data from (López-Isac et al., 2016; Yang et al., 2010). (C) Single blastomere genotyping analysis of human 2PN embryos for the induction of protective mutations on *TYK2* and *WDFY4* with FNLS-YE1. (D) Molecular etiology and genetic factors associated with higher atherosclerotic cardiovascular disease risk in FH. Low density lipoprotein cholesterol (LDL-C) level, number of mutations, and additional pathogenic and/or protective genetic variation determines CAD risk level. Figure is adapted from (Miname et al., 2019; Sturm et al., 2018). (E) Illustration of the close relationshipbetween low LDL-C level or CAD risk and clinically relevant loss-of-function alleles in *PCSK9*. Figure is a visual adaption of data, from (Cohen et al., 2006). (F) Single blastomere genotyping results of human 2PN embryos injected with FNLS-YE1 for the induction of protective mutations on *PCSK9* and *ANGPTL4* loci. INT, intact. Data are presented as the mean ± SEM. Each dot in Fig. 6C and 6F represents an embryo.

We have also examined whether FNLS-YE1-mediated base conversion in human embryos shown above could be generalized to induce preventive mutations for other diseases. SLE is a typical autoimmune disease characterized by autoantibody production and multi-organ damage (Yang et al., 2010). Genetic factors are known to play an important role in this disease (Fig. 6A), with the monozygotic twin concordance rate between 20% and 59%, and the risk for siblings of affected individuals 30 times higher than that for the general population (Arnett et al., 1976; Ramos-Niembro et al., 1978; Sestak et al., 1999). As a proof-of-concept experiment, we repurposed FNLS-YE1 for inducing protective alleles in *TYK2* and *WDFY4* genes that are associated with reduced risk for SLE (Fig. 6B) (Diogo et al., 2015; Yang et al., 2010; Zhang et al., 2014), via A928V (C > T) (Diogo et al., 2015) and R1816Q (G > A) (Yang et al., 2010) conversion, respectively. Two days after injection of FNLS-YE1 and the corresponding sgRNA in 8-cell human embryos, we analyzed the editing efficiency in single blastomeres from both 3PN and 2PN embryos and found high editing efficiency (up to 90%) at the allelic level was achieved for both *TYK2* and *WDFY4* genes (Figs. 6C and S9A). For *TYK2*, 119/126 (94.4%) and 34/37 (91.9%) of blastomeres from 3PN and 2PN embryos, respectively, had at least one edited allele (Figs. 6C and S9A). Similarly high editing efficiency was found for *WDFY4* gene (Figs. 6C and S9A). We also performed targeted deep sequencing to verify the off-target sites for *WDFY4* and found no evidence for off-sites targeting (Fig. S10). Furthermore, NTS and indel analysis showed that <5% unwanted mutations were induced by FNLS-YE1 on target loci for *TYK2* and *WDFY4* genes (Fig. S9C).

FH is a hereditary disease with a high incidence of about 1 in 250 (Akioyamen et al., 2017; Nordestgaard et al., 2013). Previous studies have identified several mutations in *LDLR*, *APOB*, and *PCSK9* genes as causative factors for FH (Sturm et al., 2018). Natural loss-of-function mutations in *PCSK9* (Cohen et al., 2006) and *ANGPTL4* (Stitziel et al., 2016) resulted in notable protective effects in reduced LDL-cholesterol level and lower risk of premature coronary artery diseases (CAD) over large-population epidemiological studies (Fig. 6D and 6E). In contrast to the induction of a nonnatural loss-of-function variant W156X of *PCSK9* shown in both 3PN and 2PN embryos (Figs. 6F and S9B), we attempted to install natural loss-of-function alleles in *PCSK9* (Harper et al., 2015) (Fig. S9E) and found five of them having targetable sequence with both NGG PAM and target base within 5-to-8 editing window for FNLS-YE1 (Fig. S9F). However, none of five alleles have >10% C-to-T conversion rate after injection of FNLS-YE1 and the corresponding sgRNA in 8-cell embryos (Fig. S9F). In addition, since W428X has no NGG PAM sequence, we thus used PAMless-FNLS-YE1 for editing (Fig. S9E). Our results showed 20%–50% conversion rate for loss-of-function mutation W428X (Fig. S9F). Moreover, we performed similar experiments on *ANGPTL4* gene previously identified for its protective effect on CAD by loss-of-function mutations (Figs. 6F and S9B). In both 3PN and 2PN embryos, 8-cell injection with FNLS-YE1 and the corresponding sgRNA enabled >65% targeted C-to-T substitution, resulting in W350X conversion for *ANGPTL4* (Figs. 6F and S9B). Furthermore, NTS and indel analysis showed that <10% unwanted mutations were induced by FNLS-YE1 or PAMless-FNLS-YE1 on target loci for *PCSK9* and *ANGPTL4* genes (Fig. S9D and S9G).

Together, these results indicate that FNLS-YE1 could efficiently introduce preventive alleles in *TYK2*/*WDFY4* and *PCSK9*/*ANGPTL4*, offering a potential approach to reduce susceptivity for SLE and FH, respectively.

## Discussion

Given the scale of homozygous ε4 distribution among the global population, we first explored a method for reducing the risk of AD through base editing while maintaining focus on minimizing the off-target effects. For this purpose, we used the ICSI method to prepare 2PN embryos carrying the ε4 allele from a heterozygous ε4 male identified by preimplantation genetic screening for *in vitro* fertilization. Given efficient and specific base substitution capability, base editing system is considered more suitable for treating >50% genetic diseases caused by different point mutations described in the ClinVar database (Doman et al., 2020; Komor et al., 2016; Zuo et al., 2020). In present study, we tested eight BEs at four different stages of human early embryos and demonstrated that the ε4 allele of *APOE* gene could be efficiently switched to the neutral ε3r variant by converting Arg158 to Cys158 using the FNLS-YE1 editor in 8-cell embryos. To ensure that the benefits of this approach far outweighed the risks, we dedicated a nontrivial level of effort to minimizing the off-target effects while characterizing the on-target effects of editing. Our results indicated that injection at the 8-cell stage led to higher on-target efficiency with fewer bystander edits or indels than other stages, possibly attributable to the less compact structure of chromatin at this stage than in earlier stages (Lee et al., 2014). Furthermore, by performing stringent control measures, our study also provides the first demonstration that FNLS-YE1 can serve as an efficient and high fidelity BE for C-to-T base conversion in human embryos. Previous human and animal studies indicated that *APOE4* clearly accelerates Aβ aggregation, resulting in the occurrence and development of AD (Lin et al., 2018; Shi et al., 2017). Thus, it is reasonable to propose that *APOE4*-targeted embryonic editing could serve as a first-line approach for homozygous ε4 couples to reduce AD susceptibility in their newborns prior to birth. Furthermore, in consideration of guidelines proposed by the National Academy of Sciences and Medicine for future potential clinical applications of gene editing in humans, our results show that this approach is highly versatile in its modification of different genes, and can be used to effectively ensure the preservation of both widely distributed or rare beneficial alleles, as appropriate for different diseases.

It is now clear that reducing plaques does not halt disease progression even in relatively early AD patients. This may be one reason why most clinically tested vaccines and mAbs targeting plaques have been ineffective thus far (Ostrowitzki et al., 2012; Rinne et al., 2010; Sevigny et al., 2016; van Dyck, 2018). In addition, preclinical experiments suggest that preventive rather than therapeutic vaccination may be more effective for limiting AD development (Petrushina et al., 2017). This finding is unsurprising if we consider that removal of plaque deposits simply leaves a hole in the nervous circuits, which is difficult damage to repair. Hence, instead of plaque removal, then prophylactic prevention of plaque accumulation in the first place may represent a more sound strategy against AD.

Further evidence that vaccine-based immunological stimulation against plaques is ineffective for treating dementia can be found in the Phase II/III clinical trial (i.e., the Generation Study) of the Novartis CAD106 vaccine (NCT02565511), which was terminated early. Designed to test if immunotherapy could prevent AD in cognitively healthy, homozygous carriers of the *APOE4* allele, aged 60–75, unexpected changes were observed in cognitive function, brain volume loss, and body weight loss. Thus, bypassing the need for a heightened immune response among elderly patients (who typically have higher rates of compromised immunity), we propose that, presuming the elimination of off-target effects in future applications, *in utero* personalized gene therapies or early-stage somatic editing approaches targeting AD-susceptible *APOE4* should result in AD-neutral *APOE3*, the phenotypes of which are well established in children and adults.

In addition to the ε2, ε3, and ε4 alleles resulting from two different C-to-T SNP haplotypes, a fourth uncommon haplotype, ε3r, has been identified in four families worldwide (Seripa et al., 2011). Although several published studies have proposed that it shares an identical phenotype with that of ε3 (Seripa et al., 2007, 2011), the rarity and physiological role of ε3r remains unknown. Since the ε3 allele carrying the R112C mutation in *APOE* is more prevalent than ε3r in our population, we focused on identifying a gene editor that was most effective at converting the ε4 allele into ε3. In addition, recent studies have reported a coding mutation (A673T) in the *APP* gene that protects against AD and cognitive decline in the elderly without AD, possibly by reducing the formation of amyloidogenic peptides (Jonsson et al., 2012). However, we observed no or very low C-to-T conversion in tests of various sgRNAs in *APOE* (112R) and APP loci (Fig. S6C and S6D), respectively. Therefore, to exploit this potentially prevalent or protective allele, it is necessary to optimize the current base editing system or use a prime editing system (Anzalone et al., 2019).

We also demonstrated FNLS-YE1 could introduce known protective variants in human embryos to potentially reduce human susceptivity to FH and SLE. However, FDA-approved drugs for FH and weak association of gene variants for SLE protection limit the advantage of inducing protective mutations by human embryonic editing. Therefore, more studies are necessary for these diseases before using human embryonic editing to introduce protective mutations.

In summary, our results firstly demonstrated that base editing with FNLS-YE1 can efficiently introduce known preventive variants without off-target effect in 8-cell human embryos, a potential approach for reducing human susceptibility to AD or other genetic diseases. Based on the increase of efficiency and specificity, we believe FNLS-YE1-mediated disease-preventive mutations can guide the development of somatic gene therapy for some genetic-based diseases.

## Materials and methods

### Ethical statement

The regulatory framework surrounding the use of human gametes and embryos in this research was based on the Management of Human Assisted Reproductive Technology (2001), Regulations of Human Assisted Reproductive Technology (2003), Human Biomedical Research Ethics Guidelines (set by National Health and Family Planning Commission of the People’s Republic of China on 1 November 2020), the Human Embryonic Stem Cell Research Ethics Guidelines (2003), the 2021 Guidelines for Stem Cell Research and Clinical Translation (issued by the International Society for Stem Cell Research, ISSCR), the latest Heritable Human Genome Editing Report (issued by the International Commission on the Clinical Use of Human Germline Genome Editing on 3 September 2020), the Helsinki Declaration, and other laws and regulations. This study was peer-reviewed with deliberating consideration given to the use of gene editing technology in human embryos for basic research by Institutional Review Board of the Shanghai Jiao Tong University Renji Hospital Assisted Reproductive Ethics Committee (RHAREC). The committee comprised of seven members from internal and external sources: a committee director/clinician, a clinical geneticist, an ethics expert, a legal expert, a director nurse, and two director doctors. Upon completion of the review, the committee approved this novel research (research license number 2017112406) using gene editing technology under the condition of strictly complying with current guidelines and policies in China. The approved study was guided by the genetic expert and monitored regularly by the RHAREC since the license was granted.

Before oocyte retrieval, all donors understood and signed the informed consent for donation of gametes for scientific research. All details of the proposed research project were clearly presented to donors, including the use of gene editing tools on gametes, embryos, and their derivatives (human embryonic stem cell lines, hESCs) to evaluate the safety and efficacy. In accordance with internationally accepted standards that developmental progression should not exceed 14 days, all human embryos used in this project were cultured *in vitro* for no >6 days, and then the subsequent genotyping analysis and the derivation of hESC were performed. Additionally, donors were informed that the donated gametes would not be used for other purposes, including but not limited to, conception for other people by assisted reproduction methods without informed consent of the study participants.

### Derivation and culture of embryonic stem cells from human embryos

A total of four CBE-edited stem cell lines and one wild-type stem cell line from oocyte donors (age 25–35 at donation) and sperm from the same donor, were obtained and used for experiments to determine on-target efficiency and off-target effects after FNLS-YE1 mediated C-to-T conversion of the *PCSK9* allele. hESCs were derived from 8-cell injected and un-injected embryos after trophectoderm biopsy and placing the inner cell mass into a 4-well plate as previously described (Yamada et al., 2014). Briefly, mural trophectoderm was ablated using a laser-assisted method with pulses of 400 ms, at 100% intensity (Hamilton Thorne; Chen et al., 2009). This method spares polar trophectoderm, which usually results in trophectoderm growth that is then ablated with additional pulses. Derivation was performed on irradiated mouse embryonic fibroblasts in knockout-Dulbecco’s modified Eagle’s medium (KO-DMEM, Gibco) with 25% knockout-serum replacement (KO-SR) and 10 mmol/L Rock inhibitor Y-27632, 10 ng/mL basic fibroblast growth factor, and 2% ES grade fetal bovine serum (FBS). Subsequently, hESCs were cultured in mTeSR1 (Stem Cell Technologies) with feeder- and serum-free conditions on growth factor-reduced Matrigel-coated dishes (Corning). When the confluence reached 70%, cells were passaged at a ratio of 1:5, or cryopreserved in a freezing solution containing 50% medium, 40% FBS (Gibco), and 10% dimethyl sulfoxide (DMSO, Sigma Aldrich). Cells were dissociated into a single-cell suspension using TrypLE (Gibco) for 3 min at 37°C, and plated into media containing Rock inhibitor Y-27632; media was replaced within 24 h. Ultimately, cells were harvested from the 6-cm dish, and genomic DNA was extracted with the DNeasy^®^ Blood & Tissue Kit (Qiagen) for WGS analysis. Furthermore, all stem cell lines underwent routine mycoplasma screening and karyotyping.

### Gametes retrieval and *in vitro* fertilization (IVF)

Semen samples were collected by masturbation after 3−5 days of abstinence. Semen was kept at 37°C for 30 min for liquefaction, followed by an established density-gradient separation method. Briefly, after the second centrifugation, the pellet was resuspended with 0.5 mL of G-IVF (Vitrolife) and incubated for standard swim-up for 30 min. The supernatant was used for insemination. For the acquisition of oocytes, cumulus-corona oocyte complexes were isolated from the follicle fluid accurately and rapidly, and then cultured in G-IVF for 3 h. Next, each oocyte was inseminated in 4-well plates with approximately 100,000 motile spermatozoa. Approximately 18–20 h after fertilization, clinically abandoned three pronuclei (3PN) embryos were collected for experiments.

### Sperm cryopreservation and thawing

Semen samples were collected by masturbation from donors into sterile containers after 3–5 days of sexual abstinence and left to liquefy at 37°C. Semen samples were placed in 15 mL centrifuge tubes, diluted 1:1 with cryoprotectant in a slow drop-wise manner, and gently mixed to form a homogeneous solution. The homogeneous solutions of semen and cryoprotectant were equally aliquoted into 0.25 mL straws. Then, the straws were placed 4 cm (−170ºC to −180ºC) above the surface of liquid nitrogen for 15 min before being transferred into liquid nitrogen for preservation.

Straws containing homogeneous solution of semen and cryoprotectant were warmed in a water bath at 37°C for 1 min. The solution was then washed with 1 mL prewarmed G-MOPS medium at least twice. The mixture was centrifuged at 360 ×*g* for 5 min to discard the supernatant, and then the pellet was resuspended in 100 μL G-MOPS medium, and stored at 37°C until it was used for intracytoplasmic sperm injection (ICSI).

### Generation of 2PN embryos by ICSI

Immature metaphase I (MI) oocytes were collected from patients for IVF or ICSI treatment. All oocytes were retrieved with informed consent from the donor or patient. MI oocytes were cultured in a maturation medium (TCM199 + 10% FBS + 10 μg/mL sodium pyruvate + 10 μg/mL FSH + 5 μg/mL LH + 1 μg/mL E2 + 1 nmol/L melatonin) until the first polar body appeared. Mature metaphase II (MII) oocytes were placed into a 50 µL micromanipulation droplet of HTF (modified human tubal fluid) with HEPES 10% buffer solution. The droplet was covered with tissue culture oil, and the dish was then mounted on the stage of an inverted microscope (Olympus IX73) equipped with a stage warmer and Narishige micromanipulators. Oocytes were fertilized by ICSI using frozen and thawed sperm. Fertilization was determined approximately 18 h after ICSI by noting the presence of two pronuclei and extrusion of the second polar body.

### *In vitro* transcription *of* CBE-variant mRNA and sgRNA

The pX330 and pCMV-CBE-variant plasmids were constructed in our lab. The pCMV-CBE-variant plasmids were linearized, purified, and the template was *in vitro* transcribed using the mMESSAGE mMACHINE T7 Ultra kit (Life Technologies). T7 promoter and sgRNA target sequence were added to sgRNA template by PCR amplification of pX330, using primers listed in Table S3. The T7-sgRNA PCR product was purified, and the template was *in vitro* transcribed using MEGA shortscript T7 Kit (Life Technologies). The sgRNA and CBE-variant mRNAs were purified using the MEGAclear kit (Life Technologies) and eluted in RNase-free water. *In vitro* transcribed RNAs were aliquoted and stored at −80°C until use. Prior to microinjection, the CBE-variant mRNA and sgRNA mixture was prepared by centrifuging for 10 min at 14,000 rpm at 4°C and then transferring the supernatant to a fresh 0.2 mL PCR tube for injection.

### CBE microinjection of human 3PN or 2PN embryos

For 1-cell injection, the mixture of CBE-variant mRNA (100 ng/μL) and sgRNA (50 ng/μL) was injected into the cytoplasm of zygotes 12–24 h after fertilization using a FemtoJet microinjector (Eppendorf) with constant flow settings. For 2-cell, 4-cell, and 8-cell injection, the mixture of CBE-variant mRNA (100 ng/μL) and sgRNA (50 ng/μL) was injected into every blastomere of the 2-cell, 4-cell, and 8-cell embryos about 30, 40, or 60 h after fertilization respectively. The injected embryos were cultured in drops of preequilibrated Global medium (LifeGlobal) under the conditions of 37°C, 6% CO_2_, 5% O_2_, and 89% N_2_. Genotyping analysis and hESCs derivation were performed 2 or 6 days later respectively.

### Single blastomere genotyping analysis

About 48 h after injection, zonae pellucidae from 8 to 16 cell stage embryos were removed by brief exposure to acidic Tyrode solution (Sigma Aldrich). Zona-free embryos were briefly (30 s) exposed to the 0.05% Trypsin-EDTA solution (Gibco) before manual disaggregation into single blastomeres with a small bore pipette. Subsequently, individual blastomeres were placed into PCR tubes with 2.5 μL embryo lysis buffer (0.1% Tween-20, 0.1% Triton X-100, 20mg/mL proteinase K, and ddH_2_O, 1:1:3:5) and incubated at 56°C for 30 min, followed by heat inactivation at 95°C for 10 min. PCR amplification was performed using nested primer sets and Phanta Max Super-Fidelity DNA Polymerase (Vazyme Biotech Co., Ltd). The first round PCR program was set as follows: 95°C for 30 s, 60°C for 30 s, and 72°C for 30 s, with a final extension at 72°C for 5 min. The second round PCR was performed using 0.5 μL PCR product as the template and nested inner PCR primers, and carried out with the same program as the first round. The PCR product was analyzed by Sanger sequencing or targeted deep sequencing to detect efficiency of base editing. It was regarded as homozygous (0% or 100% editing efficiency) for wildtype or edited genotypes when sequencing results for each blastomere detected only one type of allele. Moreover, it was regarded as heterozygous (33.3% or 66.7% editing efficiency in 3PN embryos and 50% editing efficiency in 2PN embryos) for edited genotypes when sequencing results for each blastomere detected two types of allele. PCR primers for genotype analysis are indicated in Table S3.

### Off-target analysis with targeted deep sequencing

About 50–60 h after ICSI, half of the 8-cell embryos were injected with a mixture of *GFP* mRNA, FNLS-YE1 mRNA and sgRNA, and the other half were not injected as a control. Individual GFP^+^ and GFP^-^ blastomeres were transferred into 0.2 mL PCR tubes containing 4 µL PBS and placed into a freezer at −80°C until further use. Whole genome amplification of the blastomeres was then performed using the REPLI-g Single Cell Kit (Qiagen). Briefly, samples frozen at −80°C were thawed and transferred into PCR tubes containing reconstituted buffer D2 (7 μL), and then incubated at 65°C for 10 min before the addition of stop solution (3.5 μL) and master mix (40 μL), followed by incubation at 30°C for 8 h. The DNA preparation was diluted with ddH_2_O (1:30), and 1 μL of the diluted DNA was used for PCR analysis and targeted deep sequencing.

Target sites were amplified by nested PCR from genomic DNA using Phanta Max Super-Fidelity DNA Polymerase (Vazyme Biotech Co., Ltd). The paired-end sequencing of PCR amplicons was performed with GENEWIZ Co., Ltd using the NovaSeq 6000 platform. The sequencing data were subsequently demultiplexed using fastq-multx (v1.4.1) with the PCR primers. Next, sequence alignment was performed between the demultiplexed sequencing data with each of the on- and off-target sites using CRISPResso2 (v2.0.32), and mapping statistics was generated using in-house scripts with Perl (v5.26.2) and R (v4.1.0).

Potential off-target sites of sgRNAs were predicted as previously reported (Bae et al., 2014). We extracted the top off-target sites with no >3 mismatches for *PCSK9* and *APOE* sgRNA. We then mapped the nonsynonymous SNVs and frameshift indels to the predicted off-targets by chromosome positions. PCR primers for off-target analysis are indicated in Table S3.

### Single-cell RNA sequencing analysis

Individual human blastomeres were manually picked, lysed, and subjected to one-step cDNA synthesis and amplification following the Smart-seq^®^ HT instructions (Takara). The sequencing library was constructed (Vazyme Biotech Co., Ltd), quality checked and sequenced with paired-end 150-bp reads on an Illumina NovaSeq 6000 platform. After quality checks of all libraries, 77 single-cell libraries passed our criteria and were subjected to deep sequencing. Raw reads of single-cell RNA-seq data were first trimmed and aligned to the GRCh38 human transcriptome (STAR v2.7.4a, two-pass mode). After de-duplication, RNA SNVs from individual cells were identified using the GATK pipeline (v4.2) for discovering variants in RNA-seq data. Briefly, the alignments spanning introns were first reformatted by *SplitNCigarReads*. Base quality was then re-calibrated using BaseRecalibrator and SNVs were called using *HaplotypeCaller*. Those SNVs detected in single cells with read depth ≥ 20.0, Fisher strand values ≤ 30.0, RMS mapping quality ≥ 20, and quality by depth values ≥ 2.0 were retained for downstream analysis. Further filtering was done by removing the SNV sites that overlapped with the dbSNP (v150) database downloaded from NCBI.

As the mRNAs were converted into cDNA before sequencing, both the nucleotide and its complementary base could be sequenced. For example, if there is a C in the mRNA, the cDNA has both C and G at the specific site. When the reference genome was C, the sequence would be read as C, and if the reference was G at the site, G would be read. Therefore, we counted the sum of C to T + G to A mutations for the editing of FNLS-YE1 and FNLS-BE3. Any high confidence variants found in un-injected blastomeres in each 2PN embryo were considered to be SNPs and were filtered out from the only GFP, FNLS-YE1/GFP-injected, and FNLS-BE3/GFP-injected groups for off-target analysis. The editing rate was calculated as the number of mutated reads divided by the sequencing depth for each site. Gene expression was quantified as log_2_ (reads per kilobase of transcript per million mapped reads (RPKM) + 1) using HTSeq (v0.13.5).

### WGS analysis

WGS was carried out using Illumina NovaSeq 6000 at mean coverage of 100×–150×. Trimmomatic (v0.39) was used to trim low quality reads and adapter sequences from the FASTQ files. Qualified reads were mapped to the human mitochondrial reference genome (hg19) by BWA (v0.7.12) with mem –M, and Picard-tools (v2.3.0) was used to reorder, sort, add read groups, and mark duplicates of the aligned BAM files. Then, Mutect2 (v4.1.5), Lofreq (v2.1.2), and Strelka (v2.7.1) were run separately on the aligned BAM file of hESCs for *de novo* SNVs detection, with blood samples from both parents as control. Whole genome *de novo* indels were detected using Mutect2 (v4.1.5), Scalpel (v0.5.3), and Strelka (v2.7.1) in the same way (Cibulskis et al., 2013; Fang et al., 2016; Saunders et al., 2012; Wilm et al., 2012). For example, if the WT allele is G at a certain coordinate, the hESC carries A, and blood samples from both parents carry G, then the mutant A will be called a *de novo* mutation. In contrast, if blood samples from both parents carry A, then the mutant could not be identified.

Briefly, Strelka was first used to identify the genome-wide SNVs and Indels. The regions 200 bp upstream and downstream of the variants identified by Strelka were treated as candidate regions. Second, Lofreq and Mutect2 were separately used to calculate SNVs of the candidate regions identified by Strelka. Scalpel and Mutect2 were then separately used to identify the indels of the candidate regions. The adjacent 400 bp regions of these variants were also used as candidate regions for Indel detection by Scalpel. Only variants identified by all three algorithms and with >10% allele frequencies were used for the following analysis. To strictly control the quality of the variants, we removed variants that overlapped with UCSC repeat regions or that were reported in the dbSNP151 database.

### Primer extension-mediated sequencing (PEM-seq) analysis

HEK293T cells were seeded in 10-cm dishes and cultured in DMEM supplemented with 10% FBS (Thermo Fisher Scientific) and penicillin–streptomycin at 37°C with 5% CO_2_. Cells were transfected with 10–20 μg plasmids (pU6-*PCSK9*/*APOE*/*WDFY4*-sgRNA-pCMV-FNLS-YE1-pCMV-EGFP and pU6-*PCSK9*/*APOE*/*WDFY4*-sgRNA-Cas9-pCMV-EGFP) using Lipofectamine 3000 (Thermo Fisher Scientific). Two days after transfection, cells were digested with 0.05% trypsin (Thermo Fisher Scientific) and prepared for FACS. GFP-positive cells (5 × 10^6^) were sorted and kept in DMEM to extract genomic DNA for PEM-seq analysis. Clonal H9 hESCs and ES-control1 were cultured in mTeSR1 medium (STEMCELL technology) at 37°C in a humidified atmosphere containing 5% CO_2_. Upon reaching 70% confluency, cells were dissociated into single-cell suspension using TrypLE (Gibco) for 3 min at 37°C, and 10 μg of plasmid constructs were delivered into single cells (1 × 10^6^–5 × 10^6^) by electroporation (Lonza, Nucleofector^TM^ 2b) with 100 μL hPSC nucleofection buffer (Nuwacell Biotechnology Co., Ltd) and a 0.4-cm gap cuvette. To improve survival, 10 μmol/L Rock inhibitor Y27632 (Sigma Aldrich) was added to the medium coated with Matrigel and removed after transfected cells were attached. Two to three days after electroporation, 2 × 10^6^ GFP-positive hESCs were harvested and subsequently genomic DNA was extracted for PEM-seq analysis.

PEM-seq was performed as described previously (Yin et al., 2019). In brief, 20 μg genomic DNA was extracted and sonicated to about 300–700 bp. 5ʹ biotinylated primer was placed within 200 bp from the cleavage site. For primer extension, the process was performed as follows: 95°C 3 min; 95°C 2 min, T_a_ (annealing temperature) 3 min, 5 cycles; T_a_ 3 min. Then, Bst polymerase 3.0 (NEB) was added to perform primer extension: 65°C 10 min, 80°C 5 min. Excessive biotinylated primers were depleted by 1.2× AxyPrep Mag PCR Clean-Up beads (Axygen). Purified products were heated to 95°C for 5 min and then quickly chilled on ice for 5 min for DNA denaturation. Biotinylated PCR products were enriched by Dynabeads^™^ MyOne^™^ Streptavidin C1 (Thermo Fisher Scientific). Then, PCR products on Streptavidin C1 beads were washed twice with 400 μL 1× B&W buffer [1 mol/NaCl, 5 mmol/L Tris-HCl (pH 7.4), and 1 mmol/L EDTA (pH 8.0)] followed by washing with 400 μL dH_2_O. The DNA-beads complex was then resuspended with 42.4 μL ddH_2_O. In the bridge adapter ligation step, the ligation reaction was performed in 15% PEG8000 (Sigma Aldrich) with T4 DNA ligase (Thermo Fisher Scientific) at room temperature overnight. Ligation products were washed twice with 400 μL 1× B&W buffer, then the 400 μL ddH_2_O was added, and the products were resuspended with 80 μL dH_2_O. The beads-DNA complex underwent on beads nested PCR (Taq, Transgen Biotech) with I5 and I7 sequence primers for 16 cycles. Then PCR products were recovered by size-selection beads (Axygen) followed by PCR (Fastpfu, Transgen Biotech) tagged with Illumina P5 and P7 sequences. All PEM-seq libraries were subjected to WGS performed at Macrogen using an Illumina HiSeq X Ten at a sequencing depth of 300 million reads. PEM-seq analysis and off-target hotspot identification were calculated using a previously described scoring system (Yin et al., 2019).

### Statistical analysis

All statistical values are presented as means ± SEM. Differences between datasets were considered to be significant at *P* value <0.05. All statistical tests were conducted with the unpaired student’s *t*-test (two-tailed), unless otherwise stated.

## Supplementary Material

pwac043_suppl_Supplementary_Material

## Abbreviations

Aβ: amyloid-β
AD: Alzheimer’s disease
APP: precursor protein
BBB: blood–brain barrier
BE: base editor
BE3: cytosine base editor 3
DSB: double-strand break
FH: familial hypercholesterolemia
hESCs: human embryonic stem cell lines
ICSI: intracytoplasmic sperm injection
IVF: in vitro fertilization
MI: immature metaphase I
MII: mature metaphase II
NTS: nontargeted nucleotide substitution
PEM-seq: primer extension mediated-seq
PSEN1: presenilin-1
PSEN2: presenilin-2
SLE: systemic lupus erythematosus
SNVs: single nucleotide varients
WGS: whole genome sequencing

## Declarations

Whole genome sequencing data and RNA sequencing data were deposited in Sequence Read Archive (SRA): PRJNA791471 and PRJNA769201. Additional raw data for the figures in this manuscript can be obtained from the corresponding authors upon request.

Y.H.W., M.L.Z., and H.Y. jointly conceived the project. Y.H.W., M.L.Z., C.L.X., and H.Y. designed and conducted experiments. Y.H.W., C.L.X., M.X.X., J.H.Y., J.H., and L.Z. assisted with plasmids construction, PCR, and other molecular experiments. Y.H.W., Y.Z.D., Z.F.L., D.S.W., Z.G.Z., Y.Z., H.B.L., N.Y., E.W.Z., and M.L.Z. assisted with embryonic experiments. Y.H.W., C.L.X., and J.H. assisted with derivation of human embryonic stem cells and other cellular experiments. Y.S.Z., Y.H.L., and H.F. performed bioinformatics analysis. M.L.Z., Y.Z.D., W.L., C.L.X., and H.Y. co-supervised the whole project. Y.H.W., M.L.Z., Y.Z.D., W.L., C.L.X., and H.Y. wrote the manuscript.

This work was supported by Chinese National Science and Technology major project R&D Program of China (2018YFC2000101), Strategic Priority Research Program of Chinese Academy of Science (XDB32060000), National Natural Science Foundation of China (Grant Nos. 31871502, 31901047, 31925016, 91957122, 82021001, and 31922048), Basic Frontier Scientific Research Program of Chinese Academy of Sciences From 0 to 1 original innovation project (ZDBS-LYSM001), Shanghai Municipal Science and Technology Major Project (2018SHZDZX05), Shanghai City Committee of Science and Technology Project (18411953700, 18JC1410100, 19XD1424400 and 19YF1455100), Innovative Research Team of High-Level Local Universities in Shanghai (SHSMU-ZDCX20212200 and SHSMU-ZLCX20210200), International Partnership Program of Chinese Academy of Sciences (153D31KYSB20170059), Postdoctoral Science Foundation of China (2020M681417 and 2021T140684), and Sailing Program of Shanghai (21YF1453000) (to J.H.).
