# Supplementary material for: Human 8-cell embryos enable efficient induction of disease-preventive mutations without off-target effect by cytosine base editor
Source: Protein Cell. 2022 Nov 3;14(6):416–32. doi: 10.1093/procel/pwac043 (PMC10246723; doi:10.1093/procel/pwac043)
Supplement: pwac043_suppl_Supplementary_Material [file pwac043_suppl_supplementary_material.pdf]

Supplementary Materials for

Human 8-cell embryos enable efficient induction of disease-preventive mutations without off-target effect by cytosine base editor

Yinghui Wei[1,2,†], Meiling Zhang[1,4,7,†,*], Jing Hu[2,†], Yingsi Zhou[2,†], Mingxing Xue[2,†], Jianhang Yin[5], Yuanhua Liu[2], Hu Feng[8], Ling Zhou[8], Zhifang Li[8], Dongshuang Wang[4], Zhiguo Zhang[9], Yin Zhou[4], Hongbin Liu[6], Ning Yao[4], Erwei Zuo[8], Jiazhi Hu[5], Yanzhi Du[4,*], Wen Li[1,*], Chunlong Xu[3,*], Hui Yang[2,*]

This PDF file includes:

Figs. S1-S10

Tables S1-S3

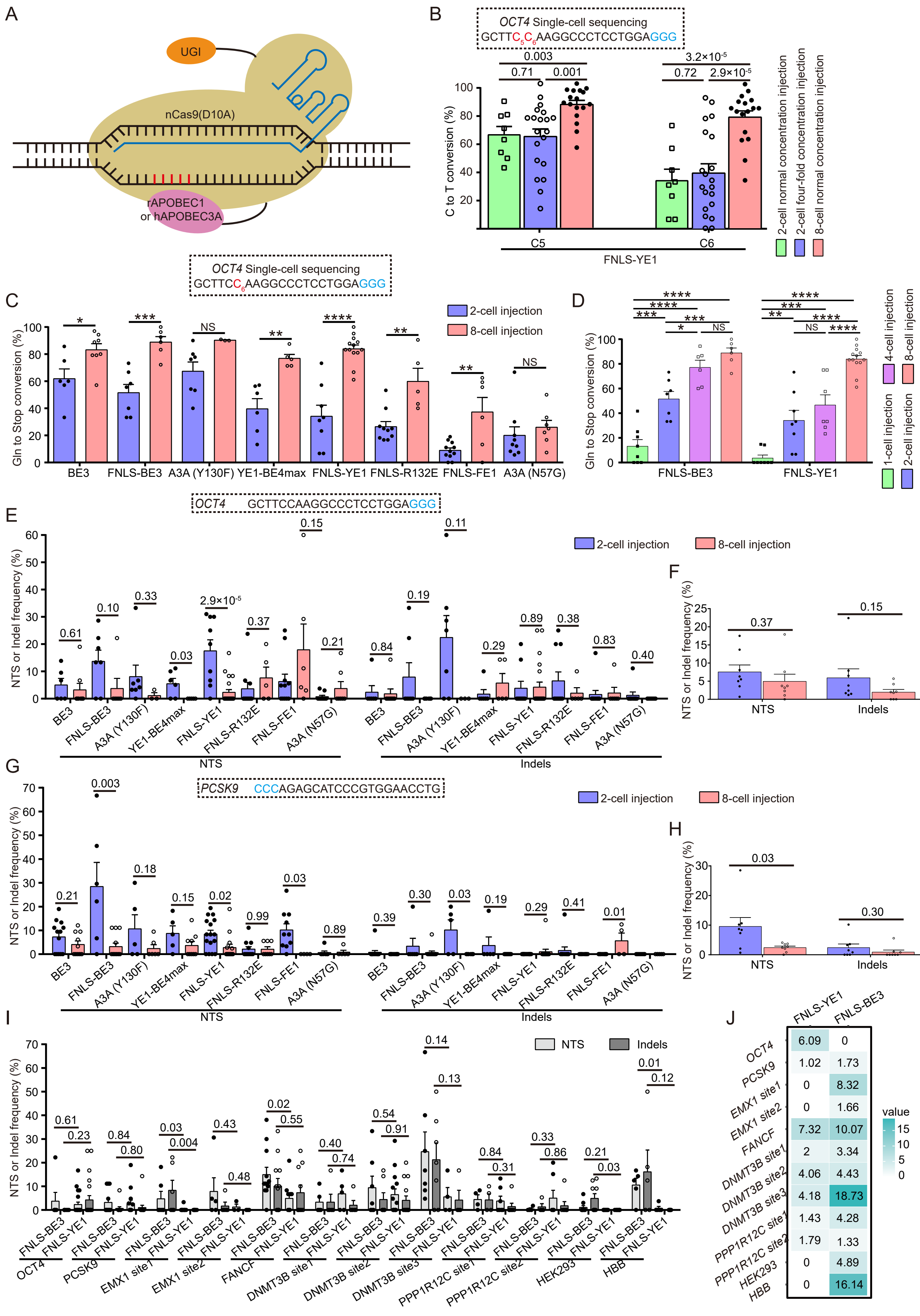

	FNLS-YE1	FNLS-BE3
OCT4	6.09	0
PCSK9	1.02	1.73
EMX1 site1	0	8.32
EMX1 site2	0	1.66
FANCF	7.32	10.07
DNMT3B site1	2	3.34
DNMT3B site2	4.06	4.43
DNMT3B site3	4.18	18.73
PPP1R12C site1	1.43	4.28
PPP1R12C site2	1.79	1.33
HEK293	0	4.89
HBB	0	16.14

Figure S1. Injection of different base editor variants in human 3PN cleavage embryos highly increased base-editing efficiency and decreased NTS or indel frequency. (A) Schematic diagram of cytosine base editor (CBE) system working principle. (B) Comparative analysis of embryos injected with different concentrations of FNLS-YE1 and sgRNA mixtures targeting the *OCT4* locus at the 2-cell and 8-cell stages. (C) Single-cell sequencing analysis of embryos injected with eight CBE variants targeting the *OCT4* locus at the 2-cell and 8-cell stages. Percentage of alleles with targeted conversion of *Gln* codon to stop codon conversion on the *OCT4* locus. (D) Single-cell sequencing analysis of embryos injected with FNLS-BE3 and FNLS-YE1 targeting the *OCT4* locus at the 1-cell, 2-cell, 4-cell, and 8-cell stages. Percentage of alleles with targeted conversion of *Gln* codon to stop codon conversion on the *OCT4* locus. (E-H) Frequency of indel mutations and non-targeted nucleotide substitutions in 2-cell and 8-cell human embryos injected with eight CBE variants targeting *OCT4* (E and F) and *PCSK9* (G and H) loci. Indels, indel mutations; NTS, non-targeted nucleotide substitutions. (I) The NTS or indel frequency of FNLS-BE3 and FNLS-YE1 targeting the 12 endogenous sites at the 8-cell stage. (J) Heatmap showing the indel distribution of embryos injected with FNLS-BE3 and FNLS-YE1 targeting human 12 endogenous sites at the 8-cell stage. Data are presented as the mean ± SEM. p values were evaluated with unpaired Student's *t*-test. $*p < 0.05$, $**p < 0.01$, $***p < 0.001$, $****p < 0.0001$; NS, not significant. Each dot in Figs. S1B-E, G, I and Figs. S1F, H represents an embryo and the base editor tested, respectively.

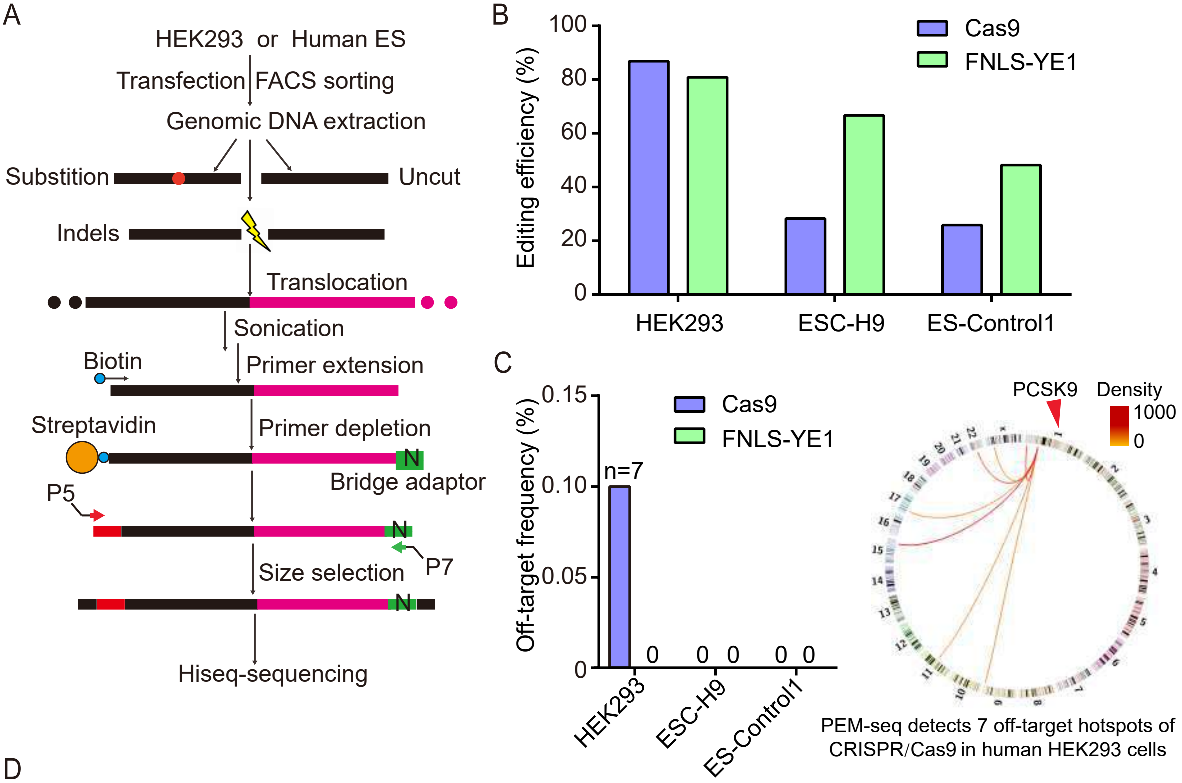

D

PEM-seq detects off-target hotspots of CRISPR/Cas9 in human HEK293 cells

Gene	No.	Target dequence	Genomic location	Direction
PCSK9	OnT	CAGGTTCCACGGGATGCTCTGGG	chr15: 55046575-55046597	-
Intergenic	OT1	CAGTTTCCACGGAATGCTCTGGG	chr15: 84580603-84580625	-
PPM1F	OT2	GCAGGCCCCCGGGATGCTTTGGG	chr22: 21946050-21946072	-
KCNAB2	OT3	CAGGCTCCACAAGATGCCCTGGG	chr1: 21946050-21946072	+
SHANK2	OT4	CCGGTTCTACAGGATGCTCTAGG	chr11: 70520931-70520953	+
Intergenic	OT5	GCAGGACCCCAGGATGCCCTGGG	chr9: 137238868-137238890	+
DMD	OT6	CGTGTTCCACGGGATGCACTTGG	chrX: 32894602-32894624	+
LRRC75A	OT7	ACAGGTCCCTGGCATGCTCTGGG	chr17: 16482410-16482432	+

E

Infromation of PEM-seq libraries in *PCSK9* locus in different cell lines

Nuclease	cell line	locus	DNA	Reads	Translocation	Indels	Germline	On-target substitions	Editing efficiency
	293T		20μg	325466	20553	262236	37064		86.9%
Cas9	ESC-H9	*PCSK9*	20μg	162249	315	45546	116056		28.3%
	ES-Control1		20μg	115280	288	29544	85156		25.9%
	293T		20μg	352378	142	2652	64595	285046	80.9%
FNLS-YE1	ESC-H9	*PCSK9*	20μg	250172	15	2267	80894	166932	66.7%
	ES-Control1		20μg	106973	14	1466	53900	51599	48.2%
	293T		20μg	478780	32	936	477815		0
Control	ESC-H9	*PCSK9*	20μg	115476	6	281	115186		0
	ES-Control1		20μg	311239	23	559	310672		0

Figure S2. Off-target profiling of Cas9 and FNLS-YE1 targeting *PCSK9* by Primer-extension-mediated sequencing (PEM-seq) in HEK293T cells and hESCs. (A) Schematic diagram of PEM-seq. See “Methods” for details. (B and C) Editing efficiencies (B) and off-target hotspots (C) detected by PEM-seq for Cas9 and FNLS-YE1 targeting *PCSK9* in HEK293T cells and hESCs. (D) List of potential off-target sites captured by PEM-seq. OnT, on-target; OT, off-target. Red letters indicate mismatched nucleotides. (E) Information of PEM-seq libraries for different samples treated with Cas9 or FNLS-YE1 targeting *PCSK9*.

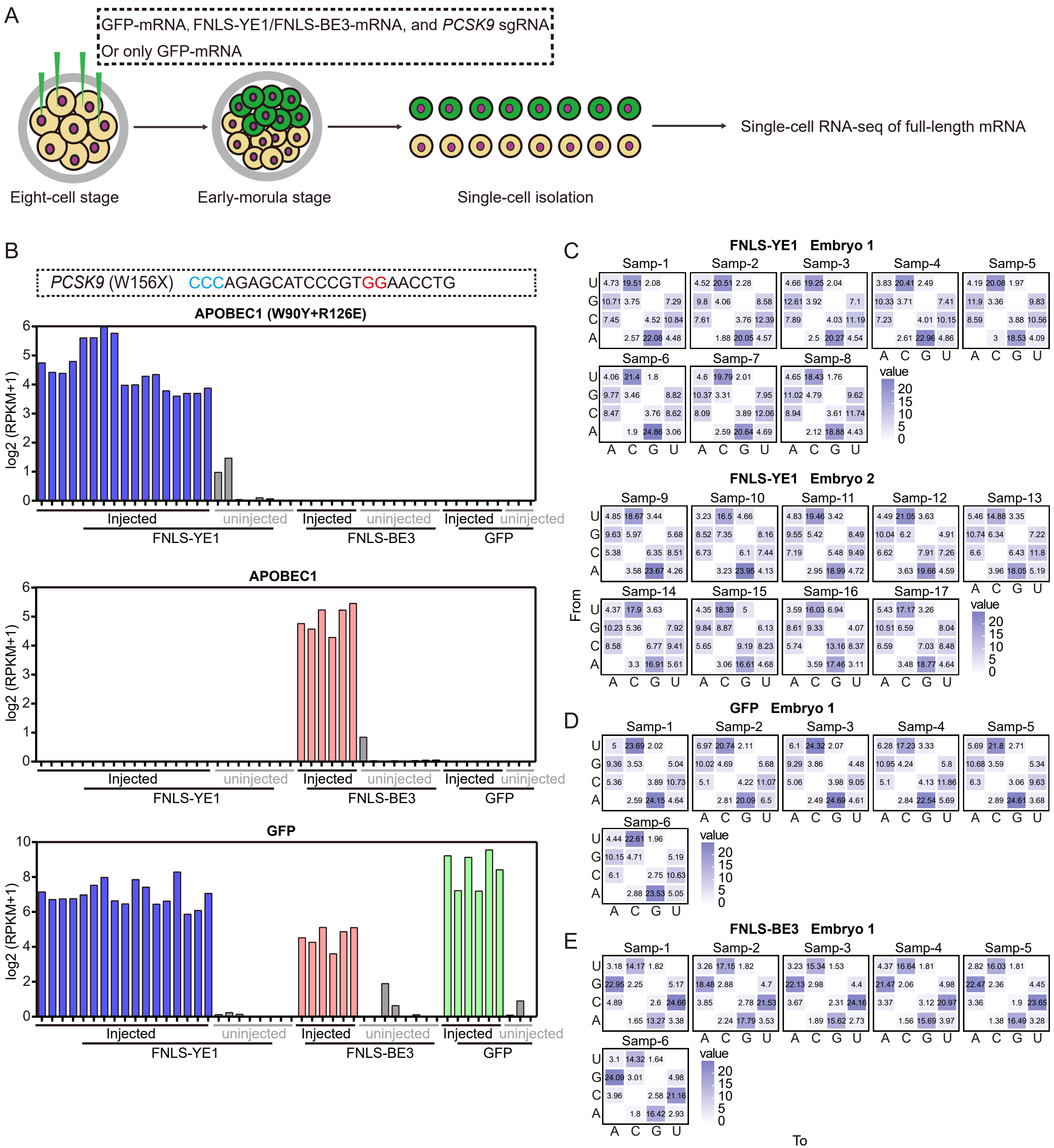
A
GFP-mRNA, FNLS-YE1/FNLS-BE3-mRNA, and PCSK9 sgRNA
Or only GFP-mRNA
Eight-cell stage
Early-morula stage
Single-cell isolation
Single-cell RNA-seq of full-length mRNA
B
PCSK9 (W156X) CCCAGAGCATCCCGTGGAACCTG
APOBEC1 (W90Y+R126E)
APOBEC1
GFP
log2 (RPKM+1)
Injected
uninjected
FNLS-YE1
FNLS-BE3
GFP
C
FNLS-YE1 Embryo 1
Samp-1: U 4.73 19.51 2.08; G 10.71 3.75 7.29; C 7.45 4.52 10.84; A 2.57 22.08 4.48
Samp-2: U 4.52 20.51 2.28; G 9.8 4.06 8.58; C 7.61 3.76 12.39; A 1.88 20.05 4.57
Samp-3: U 4.66 19.25 2.04; G 12.61 3.92 7.1; C 7.89 4.03 11.19; A 2.5 20.27 4.54
Samp-4: U 3.83 20.41 2.49; G 10.33 3.71 7.41; C 7.23 4.01 10.15; A 2.61 22.96 4.86
Samp-5: U 4.19 20.08 1.97; G 11.9 3.36 9.83; C 8.59 3.88 10.56; A 3 18.53 4.09
Samp-6: U 4.06 21.4 1.8; G 9.77 3.46 8.82; C 8.47 3.76 8.62; A 1.9 24.86 3.06
Samp-7: U 4.6 19.79 2.01; G 10.37 3.31 7.95; C 8.09 3.89 12.06; A 2.59 20.64 4.69
Samp-8: U 4.65 18.43 1.76; G 11.02 4.79 9.62; C 8.94 3.61 11.74; A 2.12 18.88 4.43
FNLS-YE1 Embryo 2
Samp-9: U 4.85 18.67 3.44; G 9.63 5.97 5.68; C 5.38 6.35 8.51; A 3.58 23.67 4.26
Samp-10: U 3.23 16.5 4.66; G 8.52 7.35 8.16; C 6.73 6.1 7.44; A 3.23 23.95 4.13
Samp-11: U 4.83 19.46 3.42; G 9.55 5.42 8.49; C 7.19 5.48 9.49; A 2.95 18.99 4.72
Samp-12: U 4.49 21.05 3.63; G 10.04 6.2 4.91; C 6.62 7.91 7.26; A 3.63 19.66 4.59
Samp-13: U 5.46 14.88 3.35; G 10.74 6.34 7.22; C 6.6 6.43 11.8; A 3.96 18.05 5.19
Samp-14: U 4.37 17.9 3.63; G 10.23 5.36 7.92; C 8.58 6.77 9.41; A 3.3 16.91 5.61
Samp-15: U 4.35 18.39 5; G 9.84 8.87 6.13; C 5.65 9.19 8.23; A 3.06 16.61 4.68
Samp-16: U 3.59 16.03 6.94; G 8.61 9.33 4.07; C 5.74 13.16 8.37; A 3.59 17.46 3.11
Samp-17: U 5.43 17.17 3.26; G 10.51 6.59 8.04; C 6.59 7.03 8.48; A 3.48 18.77 4.64
From
A C G U
value
20 15 10 5 0
D
GFP Embryo 1
Samp-1: U 5 23.69 2.02; G 9.36 3.53 5.04; C 5.36 3.89 10.73; A 2.59 24.15 4.64
Samp-2: U 6.97 20.74 2.11; G 10.02 4.69 5.68; C 5.1 4.22 11.07; A 2.81 20.09 6.5
Samp-3: U 6.1 24.32 2.07; G 9.29 3.86 4.48; C 5.06 3.98 9.05; A 2.49 24.69 4.61
Samp-4: U 6.28 17.23 3.33; G 10.95 4.24 5.8; C 5.1 4.13 11.86; A 2.84 22.54 5.69
Samp-5: U 5.69 21.8 2.71; G 10.68 3.59 5.34; C 6.3 3.06 9.63; A 2.89 24.61 3.68
Samp-6: U 4.44 22.61 1.96; G 10.15 4.71 5.19; C 6.1 2.75 10.63; A 2.88 23.53 5.05
E
FNLS-BE3 Embryo 1
Samp-1: U 3.18 14.17 1.82; G 22.95 2.25 5.17; C 4.89 2.6 24.66; A 1.65 13.27 3.38
Samp-2: U 3.26 17.15 1.82; G 18.48 2.88 4.7; C 3.85 2.78 21.53; A 2.24 17.79 3.53
Samp-3: U 3.23 15.34 1.53; G 22.13 2.98 4.4; C 3.67 2.31 24.16; A 1.89 15.62 2.73
Samp-4: U 4.37 16.64 1.81; G 21.47 2.06 4.98; C 3.37 3.12 20.97; A 1.56 15.69 3.97
Samp-5: U 2.82 16.03 1.81; G 22.47 2.36 4.45; C 3.36 1.9 23.65; A 1.38 16.49 3.28
Samp-6: U 3.1 14.32 1.64; G 24.09 3.01 4.98; C 3.96 2.58 21.16; A 1.8 16.42 2.93
To

Figure S3. RNA off-target profiling of FNLS-YE1 and FNLS-BE3 targeting *PCSK9* in human 2PN embryos. (A) Schematic of sgRNA-independent RNA off-targeting analyses using RNA sequencing of FNLS-YE1-treated embryos undergoing whole-transcriptome amplification at the single cell level. (B) Expression levels of FNLS-YE1 (APOBEC1-W90Y+R126E), FNLS-BE3 (APOBEC1), and co-injected GFP were quantified in all sequenced single blastomeres of injected embryos. (C-E) Transcriptomic SNVs distribution for different base-conversion types detected in single blastomeres of injected embryos for *PCSK9* editing.

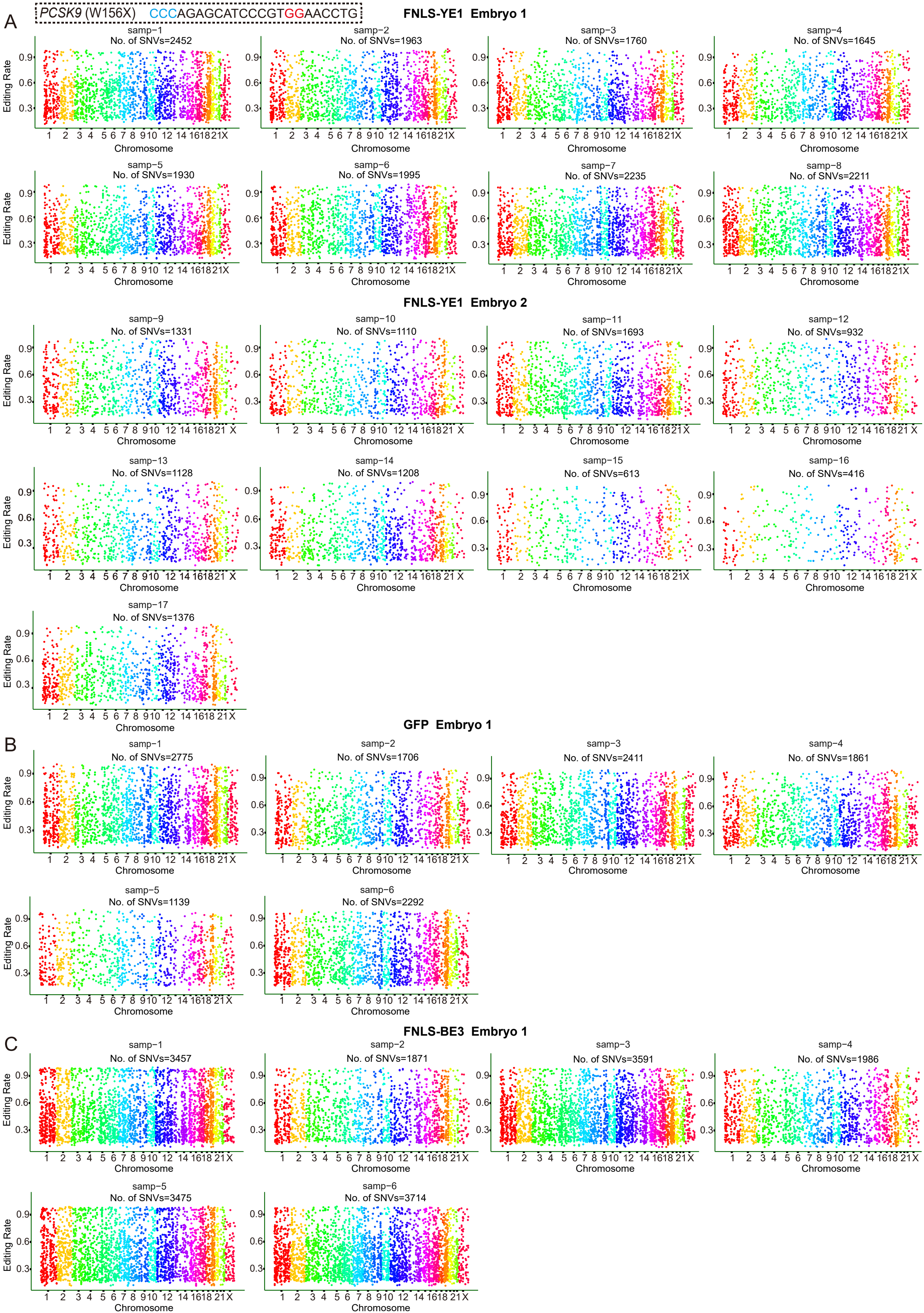
PCSK9 (W156X) CCCAGAGCATCCCGTGGAACCTG
A
FNLS-YE1 Embryo 1
samp−1 No. of SNVs=2452
samp−2 No. of SNVs=1963
samp−3 No. of SNVs=1760
samp−4 No. of SNVs=1645
samp−5 No. of SNVs=1930
samp−6 No. of SNVs=1995
samp−7 No. of SNVs=2235
samp−8 No. of SNVs=2211
FNLS-YE1 Embryo 2
samp−9 No. of SNVs=1331
samp−10 No. of SNVs=1110
samp−11 No. of SNVs=1693
samp−12 No. of SNVs=932
samp−13 No. of SNVs=1128
samp−14 No. of SNVs=1208
samp−15 No. of SNVs=613
samp−16 No. of SNVs=416
samp−17 No. of SNVs=1376
B
GFP Embryo 1
samp−1 No. of SNVs=2775
samp−2 No. of SNVs=1706
samp−3 No. of SNVs=2411
samp−4 No. of SNVs=1861
samp−5 No. of SNVs=1139
samp−6 No. of SNVs=2292
C
FNLS-BE3 Embryo 1
samp−1 No. of SNVs=3457
samp−2 No. of SNVs=1871
samp−3 No. of SNVs=3591
samp−4 No. of SNVs=1986
samp−5 No. of SNVs=3475
samp−6 No. of SNVs=3714
Editing Rate
Chromosome
1 2 3 4 5 6 7 8 9 10 12 14 16 18 21 X

Figure S4. Distribution of off-target RNA SNVs on human chromosomes for all single blastomeres of human 2PN embryos injected with only GFP, FNLS-YE1/sgRNA/GFP, or FNLS-BE3/sgRNA/GFP targeting *PCSK9*. (A) Distribution of off-target RNA SNVs on human chromosomes for FNLS-YE1/sgRNA/GFP mRNA-injected single blastomeres (n = 17 cells, two human 2PN embryos). (B) Distribution of off-target RNA SNVs on human chromosomes for only GFP mRNA-injected single blastomeres (n = 6 cells, one human 2PN embryos). (C) Distribution of off-target RNA SNVs on human chromosomes for FNLS-BE3/sgRNA/GFP mRNA-injected single blastomeres (n = 6 cells, one human 2PN embryo).

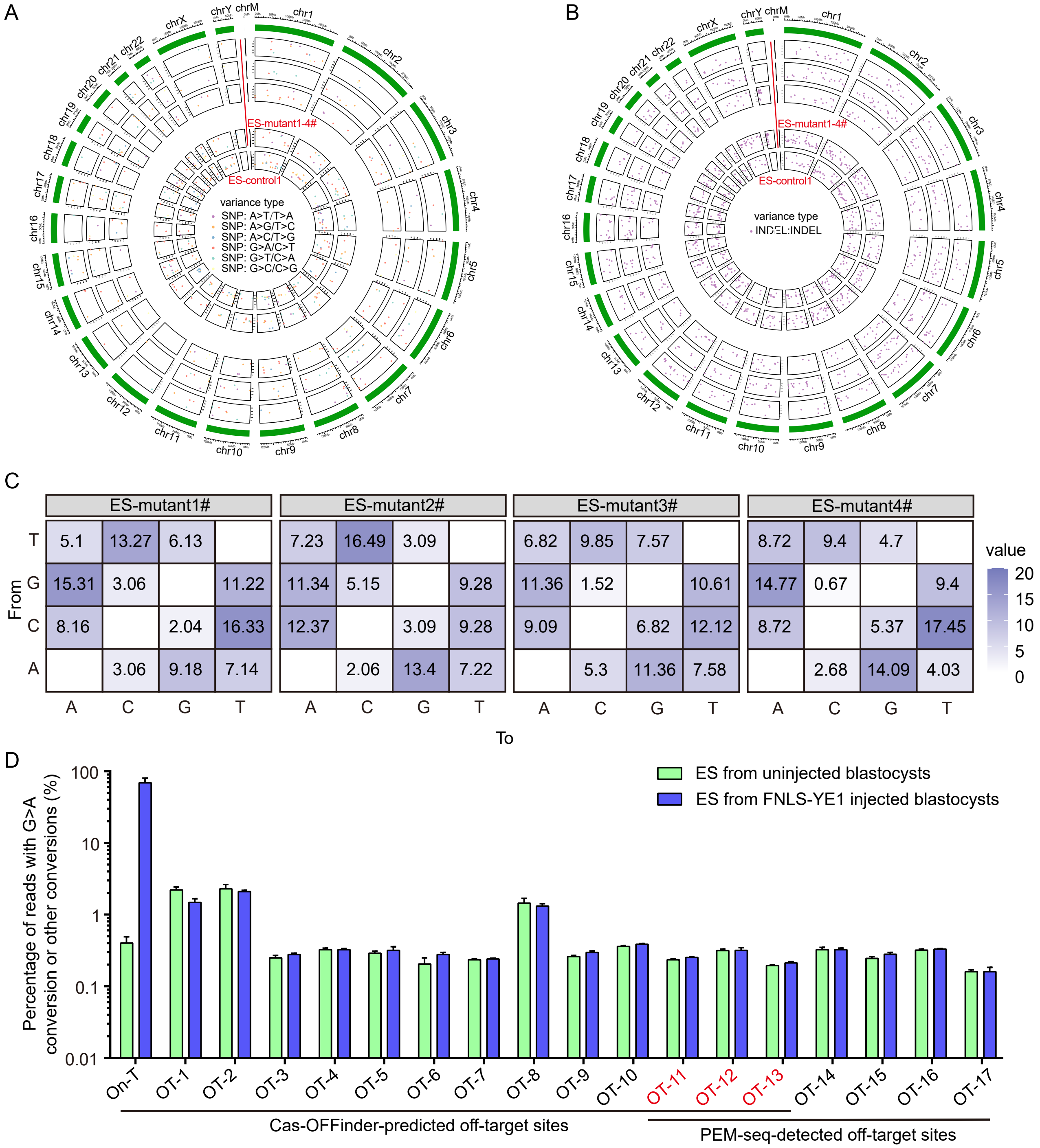

A
ES-mutant1-4#
ES-control1
variance type
SNP: A>T/T>A
SNP: A>G/T>C
SNP: A>C/T>G
SNP: G>A/C>T
SNP: G>T/C>A
SNP: G>C/C>G
B
ES-mutant1-4#
ES-control1
variance type
INDEL:INDEL
C
ES-mutant1#
ES-mutant2#
ES-mutant3#
ES-mutant4#
From
To
value
T 5.1 13.27 6.13 | 7.23 16.49 3.09 | 6.82 9.85 7.57 | 8.72 9.4 4.7
G 15.31 3.06 11.22 | 11.34 5.15 9.28 | 11.36 1.52 10.61 | 14.77 0.67 9.4
C 8.16 2.04 16.33 | 12.37 3.09 9.28 | 9.09 6.82 12.12 | 8.72 5.37 17.45
A 3.06 9.18 7.14 | 2.06 13.4 7.22 | 5.3 11.36 7.58 | 2.68 14.09 4.03
A C G T
D
ES from uninjected blastocysts
ES from FNLS-YE1 injected blastocysts
Percentage of reads with G>A conversion or other conversions (%)
100
10
1
0.1
0.01
On-T
OT-1
OT-2
OT-3
OT-4
OT-5
OT-6
OT-7
OT-8
OT-9
OT-10
OT-11
OT-12
OT-13
OT-14
OT-15
OT-16
OT-17
Cas-OFFinder-predicted off-target sites
PEM-seq-detected off-target sites

Figure S5. DNA off-target profiling of FNLS-YE1 targeting *PCSK9* in hESCs derived from non-edited and edited embryos by WGS. (A and B) Distribution of SNVs (A) and indels (B) in the human genome for control and *PCSK9*-edited hESCs. hESCs from outer circle to inner circle were ES-mutant1-4# and ES-control1. (C) Distribution frequency of SNV mutation types for *PCSK9*-edited hESCs. The number in each cell indicates the proportion of a certain type of mutation among all SNV mutations. (D) Editing rate for on-target and off-target sites in human embryonic stem cells derived from control and FNLS-YE1-treated embryos. Data are presented as the mean ± SEM.

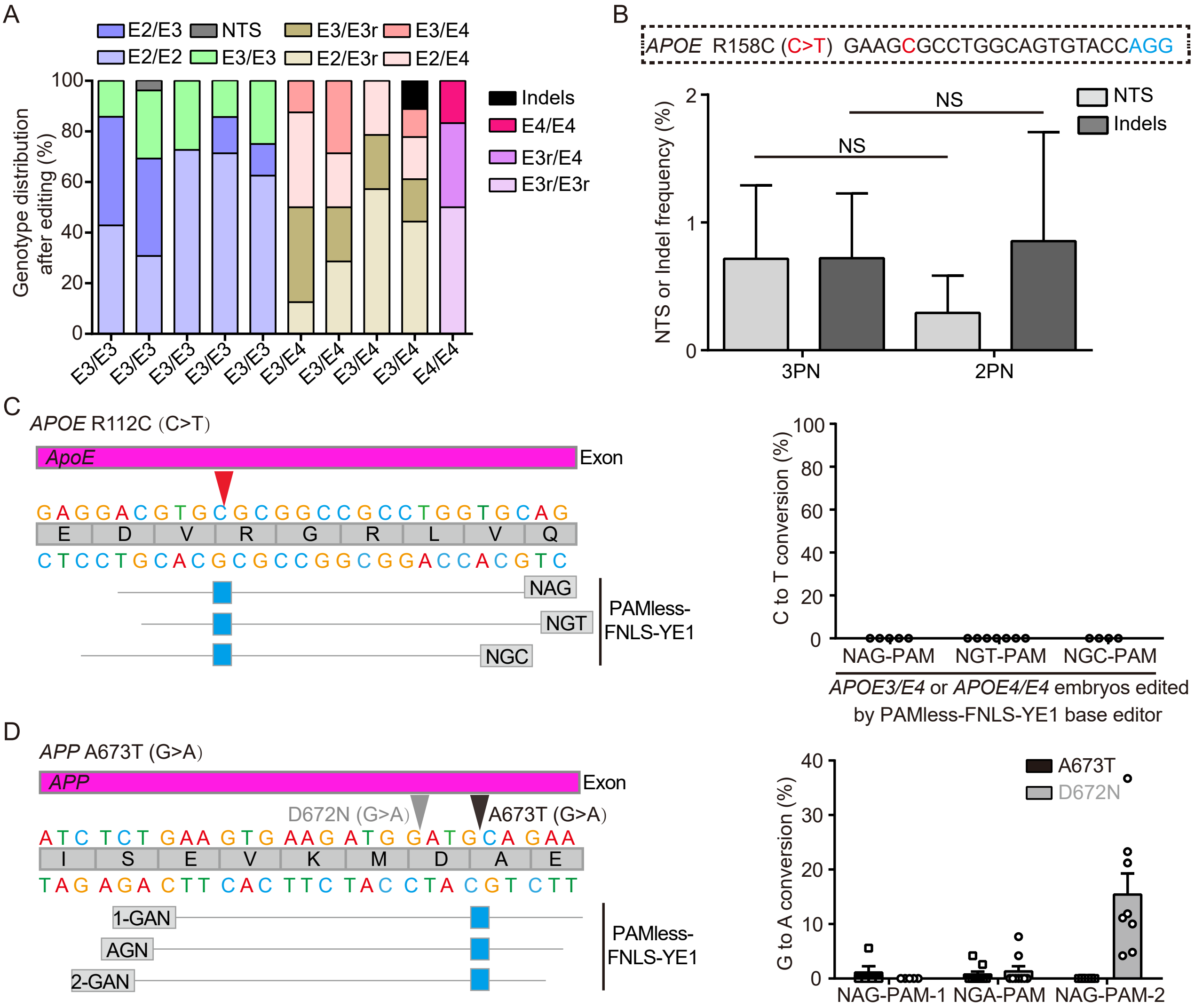
A
E2/E3 NTS E3/E3r E3/E4
E2/E2 E3/E3 E2/E3r E2/E4
Indels
E4/E4
E3r/E4
E3r/E3r
Genotype distribution after editing (%)
E3/E3 E3/E3 E3/E3 E3/E3 E3/E3 E3/E4 E3/E4 E3/E4 E3/E4 E4/E4
B
APOE R158C (C>T) GAAGCGCCTGGCAGTGTACCAGG
NTS
Indels
NS
NS
NTS or Indel frequency (%)
3PN
2PN
C
APOE R112C (C>T)
ApoE
Exon
G A G G A C G T G C G C G G C C G C C T G G T G C A G
E D V R G R L V Q
C T C C T G C A C G C G C C G G C G G A C C A C G T C
NAG
NGT
NGC
PAMless-FNLS-YE1
C to T conversion (%)
NAG-PAM NGT-PAM NGC-PAM
APOE3/E4 or APOE4/E4 embryos edited by PAMless-FNLS-YE1 base editor
D
APP A673T (G>A)
APP
Exon
D672N (G>A)
A673T (G>A)
ATC TCT GAA GTG AAG ATG GAT GCA GAA
I S E V K M D A E
TAG AGA CTT CAC TTC TAC CTA CGT CTT
1-GAN
AGN
2-GAN
PAMless-FNLS-YE1
G to A conversion (%)
A673T
D672N
NAG-PAM-1 NGA-PAM NAG-PAM-2

Figure S6. Blastomere genotyping and base conversion results from human embryos injected with FNLS-YE1 targeting *APOE4*, *APOE3*, and *APP* loci. (A) Genotype distribution after editing by FNLS-YE1 in single blastomere from human 2PN embryos carrying *APOE3/E3*, *APOE3/E4*, and *APOE4/E4*. (B) Frequency of NTS/indel formation in 3PN and 2PN embryos treated with FNLS-YE1. (C) Base conversion rate on *APOE* (R112C) locus using PAMless-FNLS-YE1. (D) Base conversion rate on *APP* (A673T) locus using PAMless-FNLS-YE1. Data are presented as the mean ± SEM. p values were evaluated with unpaired Student's *t*-test. NS, not significant. Each dot in Figs. S6C and S6D represents an embryo.

A

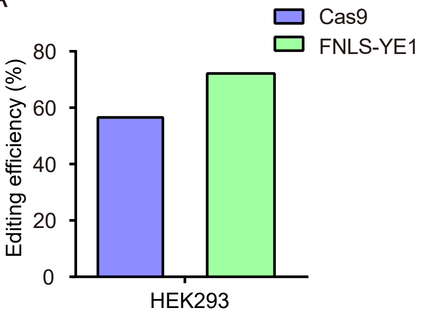

B

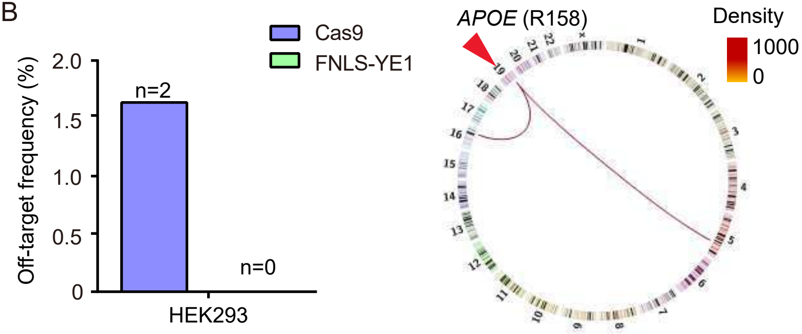

C

PEM-seq detects off-target hotspots of CRISPR/Cas9 in human HEK293 cells

Gene	No.	Target dequence	Genomic location	Direction
APOE (R158)	OnT	GAAGCGCCTGGCAGTGTACCAGG	chr19: 44908818-44908840	+
LOC105379198	OT1	AAAGCACCTAGCAGAGTACCTGG	chr5: 127219661-127219683	+
SLC9A5	OT2	AAAGTGCCTAGCAGAGTACCTGG	chr16: 67259439-67259461	+

D

Infromation of PEM-seq libraries in *APOE* (R158) locus in HEK293 cells

Nuclease	cell line	locus	DNA	Reads	Translocation	Indels	Germline	On-target substitions	Editing efficiency
Cas9	293T	*APOE*	20μg	12090	1650	5190	4420		56.6%
FNLS-YE1	293T	*APOE*	20μg	20280	40	120	14880	14640	72.2%
Control	293T	*APOE*	20μg	19250	0	80	19100		0

Figure S7. DNA off-target profiling of Cas9 and FNLS-YE1 targeting *APOE* (158R) by PEM-seq in HEK293T cells. (A and B) Editing efficiencies (A) and off-target hotspots (B) detected by PEM-seq for Cas9 and FNLS-YE1 targeting *APOE* (158R) in HEK293T cells. (C) List of potential off-target sites captured by PEM-seq. OnT, on-target; OT, off-target. Red letters indicate mismatched nucleotides. (D) Information of PEM-seq libraries for different samples treated with Cas9 or FNLS-YE1 targeting *APOE* (158R).

APOE R158C (C>T) GAAGCGCCTGGCAGTGTACCAGG

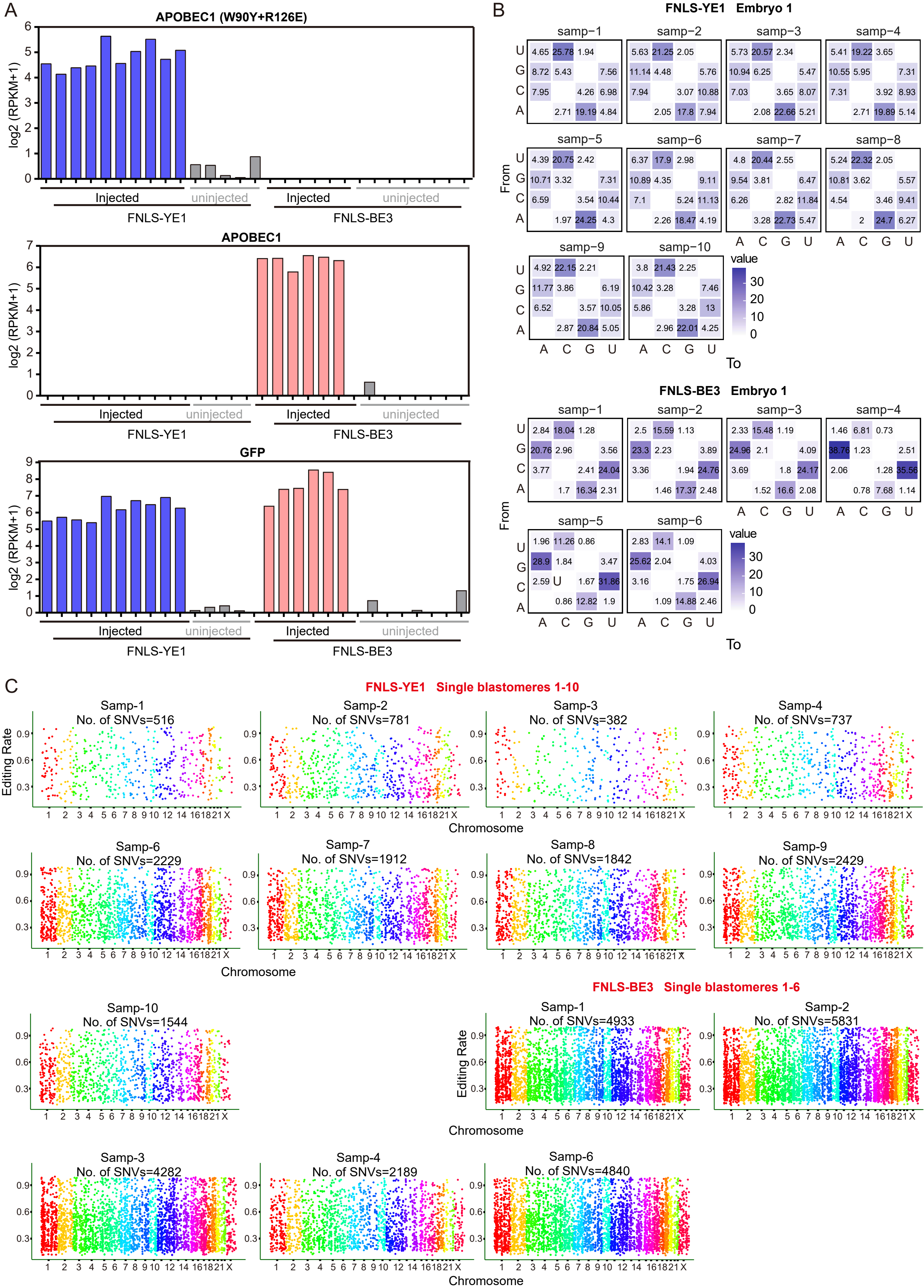

Figure S8. RNA off-target profiling of FNLS-YE1 targeting *APOE* (158R) in human 2PN embryos. (A) Expression levels of FNLS-YE1 (APOBEC1-W90Y+R126E), FNLS-BE3 (APOBEC1), and co-injected GFP were quantified in all sequenced single blastomeres of injected embryos for *APOE* (158R) editing. (B) Transcriptomic SNVs distribution for different base-conversion types detected in single blastomere of injected embryos for *APOE* (158R) editing. (C) Distribution of off-target RNA SNVs on human chromosomes for FNLS-YE1/sgRNA/GFP mRNA-injected (n = 10 cells, one human 2PN embryo) and FNLS-BE3/sgRNA/GFP mRNA-injected (n = 6 cells, one human 2PN embryos) single blastomeres.

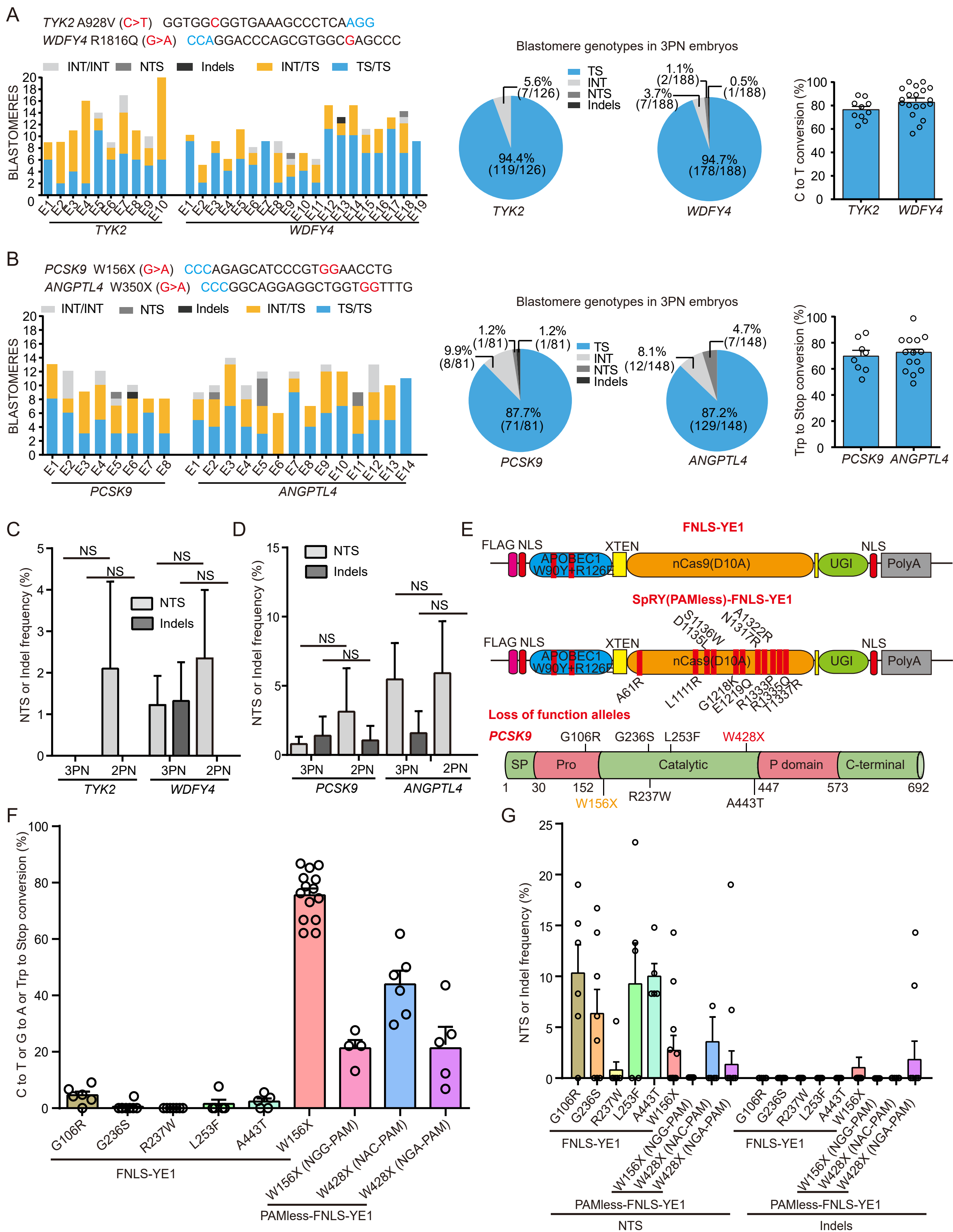
A
TYK2 A928V (C>T) GGTGGCGGTGAAAGCCCTCAAGG
WDFY4 R1816Q (G>A) CCAGGACCCAGCGTGGCGAGCCC
INT/INT NTS Indels INT/TS TS/TS
BLASTOMERES
TYK2
WDFY4
Blastomere genotypes in 3PN embryos
TS INT NTS Indels
5.6% (7/126)
94.4% (119/126)
1.1% (2/188)
3.7% (7/188)
0.5% (1/188)
94.7% (178/188)
C to T conversion (%)
B
PCSK9 W156X (G>A) CCCAGAGCATCCCGTGGAACCTG
ANGPTL4 W350X (G>A) CCCGGCAGGAGGCTGGTGGTTTG
9.9% (8/81)
1.2% (1/81)
1.2% (1/81)
87.7% (71/81)
8.1% (12/148)
4.7% (7/148)
87.2% (129/148)
PCSK9
ANGPTL4
Trp to Stop conversion (%)
C
NTS or Indel frequency (%)
NS
3PN 2PN
D
E
FNLS-YE1
FLAG NLS APOBEC1 W90Y+R126E XTEN nCas9(D10A) NLS UGI PolyA
SpRY(PAMless)-FNLS-YE1
S1136W D1135L A1322R N1317R
A61R L1111R G1218K E1219Q R1333P R1335Q T1337R
Loss of function alleles
PCSK9 G106R G236S L253F W428X
SP Pro Catalytic P domain C-terminal
1 30 152 447 573 692
W156X R237W A443T
F
C to T or G to A or Trp to Stop conversion (%)
G106R G236S R237W L253F A443T W156X
FNLS-YE1
W156X (NGG-PAM) W428X (NAC-PAM) W428X (NGA-PAM)
PAMless-FNLS-YE1
G
NTS or Indel frequency (%)
NTS
Indels

Figure S9. Efficient induction of protective mutation for systemic lupus erythematosus and familial hypercholesterolemia in human 3PN embryos. (A) Single blastomere genotyping analysis of human 3PN embryos for the induction of protective mutations on *TYK2* and *WDFY4* with FNLS-YE1 at 8-cell stage. (B) Single blastomere genotyping results of human 3PN embryos injected with FNLS-YE1 for the induction of protective mutations on *PCSK9* and *ANGPTL4* loci at 8-cell stage. (C and D) Frequency of NTS/indels formation in human 3PN embryos for *TYK2* (C), *WDFY4* (C), *PCSK9* (D), and *ANGPTL4* (D) editing with FNLS-YE1. (E) Schematics of PAMless-FNLS-YE1 and illustration of clinically *PCSK9*-related loss-of-function alleles. (F) Induction efficiency of *PCSK9*-related protective mutations in human 3PN embryos injected by FNLS-YE1 or PAMless-FNLS-YE1. (G) NTS or indels frequency of *PCSK9*-related protective mutations in human 3PN embryos injected by FNLS-YE1 and PAMless-FNLS-YE1. TS, targeted substitution; NTS, non-targeted nucleotide substitution; INT, intact; Indels, indel mutations. Data are presented as the mean ± SEM. p values were evaluated with unpaired student's t test. Each dot in Figure S9 represents an embryo.

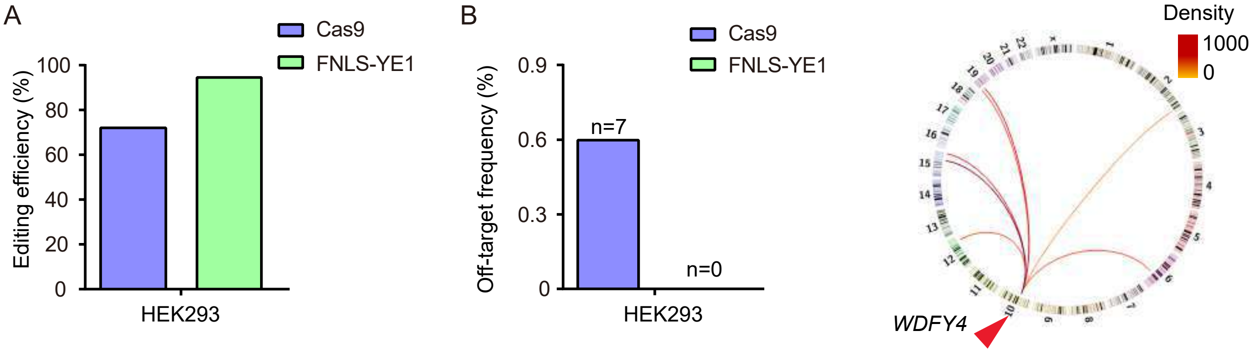

C

PEM-seq detects off-target hotspots of CRISPR/Cas9 in human HEK293 cells

Gene	No.	Target dequence	Genomic location	Direction
WDFY4	OnT	GGGCTCGCCACGCTGGGTCCTGG	chr10: 48817334-48817356	-
PIAS1	OT1	GGGCAGGCCACGCTGGGCCCAGG	chr15: 68198295-68198317	-
Intergenic	OT2	GAGCTCCCAACGCTGGGTCCAGG	chr19: 17794029-17794051	+
RASAL1	OT3	GGGCTCAGCACGTTGGGTCCAGG	chr12: 113113220-113113242	-
AL078600.1	OT4	AGGCTAGCCACTCTGGGTCCTGG	chr11: 70520931-70520953	-
CHRNG	OT5	GGGCTGGCGCCGCTGGGTCCAGG	chr2: 232542925-232542947	-
SYNM	OT6	AGGCATGTCACGCTGGGTCCCGG	chr15: 99131874-99131896	+
REXO1	OT7	GAGCTCGCCACGCTGGGAGTAGG	chr19: 1826364-1826386	+

D

Infromation of PEM-seq libraries in *WDFY4* locus in HEK293 cells

Nuclease	cell line	locus	DNA	Reads	Translocation	Indels	Germline	On-target substitions	Editing efficiency
Cas9	293T	*WDFY4*	20μg	163040	4763	112614	44785		72.0%
FNLS-YE1	293T	*WDFY4*	20μg	64679	18	355	62255	61153	94.5%
Control	293T	*WDFY4*	20μg	313183	24	1198	311945		0

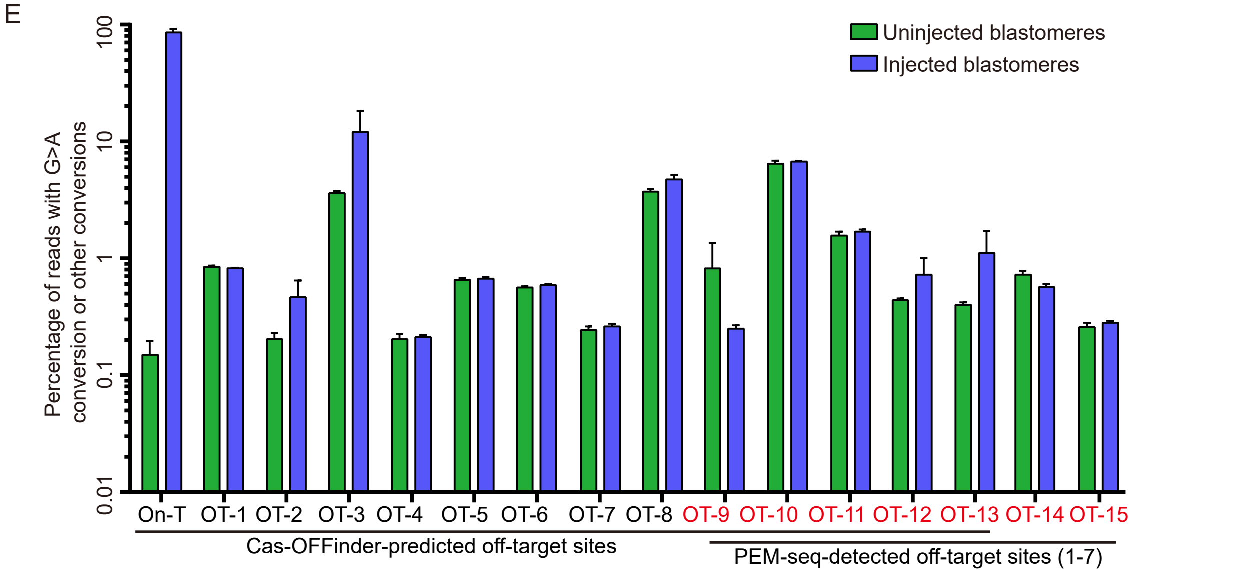

Figure S10. DNA off-target profiling of Cas9 and FNLS-YE1 targeting *WDFY4* by PEM-seq in HEK293T cells. (A and B) Editing efficiencies (A) and off-target hotspots (B) detected by PEM-seq for Cas9 and FNLS-YE1 targeting *WDFY4* in HEK293T cells. (C) List of potential off-target sites captured by PEM-seq. OnT, on-target sites; OT, off-target sites. Red letters indicate mismatched nucleotides. (D) Information of PEM-seq libraries for different samples treated with Cas9 or FNLS-YE1 targeting *WDFY4*. (E) Targeted deep sequencing analysis of the on-target and potential off-target loci predicted by Cas-OFFinder and captured by PEM-seq for *WDFY4* editing in human blastomeres injected with or without FNLS-YE1. Data are presented as the mean ± SEM.

Table S1. Karyotype and origin of ES cells derived from FNLS-YE1 microinjected embryos

ES cell line designation	Injection stage	Karyotype of ES	Karyotype of egg donor	Karyotype of sperm donor
ES-Mutant1#	Eight-cell	46, XX	46, XX	46, XY
ES-Mutant2#	Eight-cell	46, XY	46, XX	46, XY
ES-Mutant3#	Eight-cell	46, XX	46, XX	46, XY
ES-Mutant4#	Eight-cell	46, XY	46, XX	46, XY
ES-Control1	Intact control	46, XX	46, XX	46, XY

Table S2. Preimplantation development of human oocytes and zygotes after FNLS-YE1 injection

Group	No. of MI (MII) oocytes	Fertilized	Two-cells	Eight-cells	Blastocysts	Obtained ES cell lines
Intact control	28 (20)	16	13	9	5	1
Eight-cell injection	221 (163)	130	102	70	36	4

Table S3. List of primers used in this study

List of PCR primers used for generating PCR products to serve as subestrates for T7 transcription	
rev_sgRNA_T7	aaaagcaccgactcggtgcc
fwd_*OCT4*	taatacgactcactatagggcttccaaggccctcctggagttttagagctagaaatag
fwd_*PCSK9_W156X*	taatacgactcactatagggcaggttccacgggatgctctgttttagagctagaaatag
fwd_*EMX1 site1*	taatacgactcactatagggggagtccgagcagaagaagaagttttagagctagaaatag
fwd_*EMX1 site2*	taatacgactcactataggggtattcacctgaaagtgtgcgttttagagctagaaatag
fwd_*FANCF*	taatacgactcactatagggggaatcccttctgcagcaccgttttagagctagaaatag
fwd_*DNMT3B site1*	taatacgactcactatagggagtctccacacaggtgctgtgttttagagctagaaatag
fwd_*DNMT3B site2*	taatacgactcactatagggtgtccccatcctgccccaggttttagagctagaaatag
fwd_*DNMT3B site3*	taatacgactcactatagggaagtcctcctactactgcccgttttagagctagaaatag
fwd_*PPP1R12C site1*	taatacgactcactatagggggactcacccaggagtgcgttgttttagagctagaaatag
fwd_*PPP1R12C site2*	taatacgactcactatagggggagctcactgaacgctggcagttttagagctagaaatag
fwd_*HEK293*	taatacgactcactatagggggcccagactgagcacgtgagttttagagctagaaatag
fwd_*TYK2_A928V*	taatacgactcactatagggggtggcggtgaaagccctcagttttagagctagaaatag
fwd_*WDFY4_R1816Q*	taatacgactcactatagggggctcgccacgctgggtccgttttagagctagaaatag
fwd_*HBB*	taatacgactcactatagggcttgccccacagggcagtaagttttagagctagaaatag
fwd_*APOE_R158C*	taatacgactcactatagggggaagcgcctggcagtgtaccgttttagagctagaaatag
fwd_*APOE_R112C*_SpRY NAG-PAM	taatacgactcactatagggacgtgcgcggccgcctggtggttttagagctagaaatag
fwd_*APOE_R112C*_SpRY NGT-PAM	taatacgactcactatagggcgtgcgcggccgcctggtgcgttttagagctagaaatag
fwd_*APOE_R112C*_SpRY NGC-PAM	taatacgactcactatagggggacgtgcgcggccgcctgggttttagagctagaaatag
fwd_*APP_A673T*_SpRY NAG-PAM-1	taatacgactcactatagggttctgcatccatcttcacttgttttagagctagaaatag
fwd_*APP_A673T*_SpRY NGA-PAM	taatacgactcactatagggtctgcatccatcttcacttcgttttagagctagaaatag
fwd_APP_*A673T*_SpRY NAG-PAM-2	taatacgactcactatagggctgcatccatcttcacttcagttttagagctagaaatag
fwd_*ANGPTL4_W350X*	taatacgactcactatagggcaaaccaccagcctcctgccgttttagagctagaaatag
fwd_*PCSK9_G106R*	taatacgactcactatagggggtatccccggcgggcagcctgttttagagctagaaatag
fwd_*PCSK9_G236S*	taatacgactcactatagggggccgctgaccacccctgccgttttagagctagaaatag
fwd_*PCSK9_R237W*	taatacgactcactatagggcagcggccgggatgccggcggttttagagctagaaatag
fwd_*PCSK9_L253F*	taatacgactcactatagggcgtgctcaactgccaagggagttttagagctagaaatag
fwd_PCSK9_W428X_SpRY NAC-PAM	taatacgactcactataggggggaaccaggcctcattgatgttttagagctagaaatag
fwd_PCSK9_W428X_SpRY NGA-PAM	taatacgactcactatagggagggaaccaggcctcattgagttttagagctagaaatag
fwd_*PCSK9_A443T*	taatacgactcactatagggcagggcggccaccaggttgggttttagagctagaaatag
List of PCR primers used for genotyping of targeting sites	
OCT4 Genotyping OF	cttccttccccatggcggga
OCT4 Genotyping OR	atcaggctgccctgtcatga
OCT4 Genotyping IF	cacctggcttcggatttcgc
OCT4 Genotyping IR	accccgtcgaagctcactt
PCSK9_W156X Genotyping OF	cactagcagggacaaggtgg
PCSK9_W156X Genotyping OR	ttgcatttgtggggcaacag
PCSK9_W156X Genotyping IF	tgatcaggtaaggccaggct
PCSK9_W156X Genotyping OF	gaagtggaaaccaccagcag
EMX1 site1 Genotyping OF	ccactgtgtcctcttcctgc
EMX1 site1 Genotyping OR	gtttgtggttgcccacccta
EMX1 site1 Genotyping IF	ctgccatccccttctgtgaa
EMX1 site1 Genotyping IR	cccaccctagtcattggagg
EMX1 site2 Genotyping OF	agacagggaggcttagggag
EMX1 site2 Genotyping OR	tggaggagggacacctactg
EMX1 site2 Genotyping IF	gcatggcattgattgggctt
EMX1 site2 Genotyping IR	gcagaagaagggttggcttg
FANCF Genotyping OF	ggtaggatgccctacatctgc
FANCF Genotyping OR	gttcgctaatcccggaactg
FANCF Genotyping IF	agaggcgtatcatttcgcgg
FANCF Genotyping IR	ctacctgcgccacatccatc
DNMT3B site1/site2/site3 Genotyping OF	cctcaactgccaaaagccac
DNMT3B site1/site2/site3 Genotyping OR	acagctcaaggaagcgatcc
DNMT3B site1/site2/site3 Genotyping IF	gctgtttgtcttgtggcagg
DNMT3B site1/site2/site3 Genotyping IR	ggtggaggccctaggataca
PPP1R12C site1 Genotyping OF	tcaaacccctaccaactggg
PPP1R12C site1 Genotyping OR	gaacccacgcggaatcctat
PPP1R12C site1 Genotyping IF	ccctaccaactgggactgtca
PPP1R12C site1 Genotyping IR	acaaccaccccatgaagcgg
PPP1R12C site2 Genotyping OF	ctggtcacccacaaggtgt
PPP1R12C site2 Genotyping OR	agaaagtgtccccagatgctc
PPP1R12C site2 Genotyping IF	gtcatgcgtgacacgtgga
PPP1R12C site2 Genotyping IR	ctgaccctgccccacctatt
HEK293 Genotyping OF	accactgcgatatgaccacc
HEK293 Genotyping OR	taagcaagggctgatgtggg
HEK293 Genotyping IF	gcccagccaaacttgtcaac
HEK293 Genotyping IR	gctgcctagaaaggcatgga
HBB Genotyping OF	acatttgcttctgacacaactgt
HBB Genotyping OR	ggtccaagggtagaccaccag
HBB Genotyping IF	gttcactagcaacctcaaacag
HBB Genotyping IR	ccagcagcctaagggtggga
TYK2_A928V Genotyping OF	aggagtttggggggttgagga
TYK2_A928V Genotyping OR	gctcacccagatgccaagaa
TYK2_A928V Genotyping IF	gcctggggtattccgaaagg
TYK2_A928V Genotyping IR	caagtctctaggactcgccg

WDFY4_R1816Q Genotyping OF	ctcagctcctctggttaggg
WDFY4_R1816Q Genotyping OR	caatgttccgctcaagttgc
WDFY4_R1816Q Genotyping IF	tacctctcaccaagtgtgcc
WDFY4_R1816Q Genotyping IR	ccctttgcccttgaaggatga
ANGPTL4_W350X Genotyping OF	cgcagtgggtcatacctg
ANGPTL4_W350X Genotyping OR	gtcaccgtctttcgtggg
ANGPTL4_W350X Genotyping IF	gcctgggtgacagagtaag
ANGPTL4_W350X Genotyping IR	tgggatggagcggaagta
APOE_R112C/R158C Genotyping OF	ggacgagaccatgaaggagtt
APOE_R112C/R158C Genotyping OR	ggcgttcagtgattgtcgct
APOE_R112C/R158C Genotyping IF	GAATTCNNNNNNGGATCCaggcctacaaatcggaactgg
APOE_R112C/R158C Genotyping IR	GAATTCNNNNNNGGATCCctcgaaccagctcttgaggc
APP_A673T Genotyping OF	tgccaacctctcaaccaggat
APP_A673T Genotyping OR	ttctagcacaggatgaaccagag
APP_A673T Genotyping IF	aaaggcagcagaagccttactt
APP_A673T Genotyping IR	taggtcatttggcaagacaaaca
PCSK9_G106R Genotyping OF	cgagtgtggcccgtagttc
PCSK9_G106R Genotyping OR	tggccaggcttaaagggaat
PCSK9_G106R Genotyping IF	gctttttggtccgcatttggt
PCSK9_G106R Genotyping OF	gttactgtgcttggtacccga
PCSK9_G236S/R237W/L253F Genotyping OF	tcctctcccacaaatgtcgc
PCSK9_G236S/R237W/L253F Genotyping OR	agatcacctcgagtccctcc
PCSK9_G236S/R237W/L253F Genotyping IF	agcctggtggaggtgtatct
PCSK9_G236S/R237W/L253F Genotyping OF	gagcagtgtggaactgtgga
PCSK9_W428X/A443T Genotyping OF	tgcacagaggaaacacgagg
PCSK9_W428X/A443T Genotyping OR	cagctgccaacctgcaaaaa
PCSK9_W428X/A443T Genotyping IF	gaaggcatcttggaggaggg
PCSK9_W428X/A443T Genotyping OF	ggtggcgtcatcctccttac
List of PCR primers used in targeted deep sequencing of *WDFY4*_R1816Q, *PCSK9*_W156X and *APOE*_R158C off-target sites (Cas-OFFinder-predicted or PEM-seq-detected)	
WDFY4_R1816Q on target primer-OF	ctcagctcctctggttaggg
WDFY4_R1816Q on target primer-OR	caatgttccgctcaagttgc
WDFY4_R1816Q on target primer-IF (Cas-OFFinder-predicted)	GAATTCAGTTCCGGATCCtggcaggttctgaggatggt
WDFY4_R1816Q on target primer-IR (Cas-OFFinder-predicted)	tgggacagcagcatgtccta
WDFY4_R1816Q off target primer-OF site-1 (Cas-OFFinder-predicted)	acgagtatgagacaggcgct
WDFY4_R1816Q off target primer-OR site-1 (Cas-OFFinder-predicted)	tctcgaacttcaggccgtt
WDFY4_R1816Q off target primer-IF site-1 (Cas-OFFinder-predicted)	GAATTCCGATGTGGATCCcaacaagagcctcccctaac
WDFY4_R1816Q off target primer-IR site-1 (Cas-OFFinder-predicted)	aatcacgggctggacgtcgg
WDFY4_R1816Q off target primer-OF site-2 (Cas-OFFinder-predicted)	gcgtcctggagacataccag
WDFY4_R1816Q off target primer-OR site-2 (Cas-OFFinder-predicted)	atgggcaatgcaagtcacca
WDFY4_R1816Q off target primer-IF site-2 (Cas-OFFinder-predicted)	GAATTCTTAGGCGGATCCccatatggggctgctgcttg
WDFY4_R1816Q off target primer-IR site-2 (Cas-OFFinder-predicted)	atagcttccacttcatgttc
WDFY4_R1816Q off target primer-OF site-3 (Cas-OFFinder-predicted)	gaaggtccatggtggtgagg
WDFY4_R1816Q off target primer-OR site-3 (Cas-OFFinder-predicted)	agaaattgcccgacaaaccg
WDFY4_R1816Q off target primer-IF site-3 (Cas-OFFinder-predicted)	GAATTCTGACCAGGATCCagtgtctcagaggcaggtgg
WDFY4_R1816Q off target primer-IR site-3 (Cas-OFFinder-predicted)	tcacagtggaacaacagtca
WDFY4_R1816Q off target primer-OF site-4 (Cas-OFFinder-predicted)	ccaccttagcagccacgatt
WDFY4_R1816Q off target primer-OR site-4 (Cas-OFFinder-predicted)	gttttctctgaagccgctgg
WDFY4_R1816Q off target primer-IF site-4 (Cas-OFFinder-predicted)	GAATTCACAGTGGGATCCgacatcttcgtgaggacacc
WDFY4_R1816Q off target primer-IR site-4 (Cas-OFFinder-predicted)	ggttctacgtgtgaaagcaa
WDFY4_R1816Q off target primer-OF site-5 (Cas-OFFinder-predicted)	gagaatagaatggaggaatg
WDFY4_R1816Q off target primer-OR site-5 (Cas-OFFinder-predicted)	gagaatcgcttgaactgg
WDFY4_R1816Q off target primer-IF site-5 (Cas-OFFinder-predicted)	GAATTCGCCAATGGATCCgtgctgtgcgaggatgttct
WDFY4_R1816Q off target primer-IR site-5 (Cas-OFFinder-predicted)	aagaggactgccctcacttc
WDFY4_R1816Q off target primer-OF site-6 (Cas-OFFinder-predicted)	tacacccgtccagagaccaa
WDFY4_R1816Q off target primer-OR site-6 (Cas-OFFinder-predicted)	gagtctgcagcacaaaagcc
WDFY4_R1816Q off target primer-IF site-6 (Cas-OFFinder-predicted)	GAATTCACTTGAGGATCCcgccccattcatccccggga
WDFY4_R1816Q off target primer-IR site-6 (Cas-OFFinder-predicted)	aggagggtatccatgtggga
WDFY4_R1816Q off target primer-OF site-7 (Cas-OFFinder-predicted)	atccggatgctttgtgcctt
WDFY4_R1816Q off target primer-OR site-7 (Cas-OFFinder-predicted)	ttgctggcattctgggcttt
WDFY4_R1816Q off target primer-IF site-7 (Cas-OFFinder-predicted)	GAATTCTAGCTTGGATCCgattgaggcctcccaagtga
WDFY4_R1816Q off target primer-IR site-7 (Cas-OFFinder-predicted)	cttattcccaaggcatcggc
WDFY4_R1816Q off target primer-OF site-8 (Cas-OFFinder-predicted)	aaatagggaccacagctgcc
WDFY4_R1816Q off target primer-OR site-8 (Cas-OFFinder-predicted)	tggaaggactcctctctggg
WDFY4_R1816Q off target primer-IF site-8 (Cas-OFFinder-predicted)	GAATTCCTTGTAGGATCCtatttactcccactgaagcc
WDFY4_R1816Q off target primer-IR site-8 (Cas-OFFinder-predicted)	tcaaatgtccagcggtggcc
WDFY4_R1816Q off target primer-OF site-9 (Cas-OFFinder-predicted and PEM-seq-detected)	cttctagcgcttccctcctg
WDFY4_R1816Q off target primer-OR site-9 (Cas-OFFinder-predicted and PEM-seq-detected)	tggggttgctgtttgcattg
WDFY4_R1816Q off target primer-IF site-9 (Cas-OFFinder-predicted and PEM-seq-detected)	GAATTCGGCTACGGATCCaccacccctttggagctgga
WDFY4_R1816Q off target primer-IR site-9 (Cas-OFFinder-predicted and PEM-seq-detected)	ggggtcaccaccatctgcta
WDFY4_R1816Q off target primer-OF site-10 (Cas-OFFinder-predicted and PEM-seq-detected)	gaggccagtagtgccacatt
WDFY4_R1816Q off target primer-OR site-10 (Cas-OFFinder-predicted and PEM-seq-detected)	ccaggacccgaaaacctacg
WDFY4_R1816Q off target primer-IF site-10 (Cas-OFFinder-predicted and PEM-seq-detected)	GAATTCGATCAGGGATCCttcctcctcgggaagaaaca
WDFY4_R1816Q off target primer-IR site-10 (Cas-OFFinder-predicted and PEM-seq-detected)	gcacctggcctcagatttgt
WDFY4_R1816Q off target primer-OF site-11 (Cas-OFFinder-predicted and PEM-seq-detected)	atcgtcagtgttcagcccag
WDFY4_R1816Q off target primer-OR site-11 (Cas-OFFinder-predicted and PEM-seq-detected)	tgctgtccacaccttctgac
WDFY4_R1816Q off target primer-IF site-11 (Cas-OFFinder-predicted and PEM-seq-detected)	GAATTCATCACGGGATCCcagattgagggtctgtcttg

WDFY4_R1816Q off target primer-IR site-11 (Cas-OFFinder-predicted and PEM-seq-detected)	aacgggaacaagtaaggcta
WDFY4_R1816Q off target primer-OF site-12 (Cas-OFFinder-predicted and PEM-seq-detected)	tggtggaggcttctattc
WDFY4_R1816Q off target primer-OR site-12 (Cas-OFFinder-predicted and PEM-seq-detected)	gaggctttaccatgttgc
WDFY4_R1816Q off target primer-IF site-12 (Cas-OFFinder-predicted and PEM-seq-detected)	GAATTCAGTCAAGGATCCtttataacatatgtagaagt
WDFY4_R1816Q off target primer-IR site-12 (Cas-OFFinder-predicted and PEM-seq-detected)	cttcatttctcctctccctc
WDFY4_R1816Q off target primer-OF site-13 (Cas-OFFinder-predicted and PEM-seq-detected)	gaggccttcacaggtaaccc
WDFY4_R1816Q off target primer-OR site-13 (Cas-OFFinder-predicted and PEM-seq-detected)	gctatccatgaccgagaggc
WDFY4_R1816Q off target primer-IF site-13 (Cas-OFFinder-predicted and PEM-seq-detected)	GAATTCCAGATCGGATCCttcttgtgcccactcctgcc
WDFY4_R1816Q off target primer-IR site-13 (Cas-OFFinder-predicted and PEM-seq-detected)	gggcgatgatgttgatgacg
PCSK9_W156X on target primer-OF	cactagcagggacaaggtgg
PCSK9_W156X on target primer-OR	ttgcatttgtggggcaacag
PCSK9_W156X on target primer-IF	GAATTCAGTTCCGGATCCaaatggcttaagcagagtcc
PCSK9_W156X on target primer-IR	aatggattcagctcagatgg
PCSK9_W156X off target primer-OF site-1 (Cas-OFFinder-predicted)	accacgtagccagcctgtaa
PCSK9_W156X off target primer-OR site-1 (Cas-OFFinder-predicted)	gtttcccgtgaacgcaacat
PCSK9_W156X off target primer-IF site-1 (Cas-OFFinder-predicted)	GAATTCCGATGTGGATCCggcgcgcgctcggctggggt
PCSK9_W156X off target primer-IR site-1 (Cas-OFFinder-predicted)	agtccgagtgtgagcaagcc
PCSK9_W156X off target primer-OF site-2 (Cas-OFFinder-predicted)	ctggcaaaatctagagcatatggg
PCSK9_W156X off target primer-OR site-2 (Cas-OFFinder-predicted)	cttcatggtgctttctggcac
PCSK9_W156X off target primer-IF site-2 (Cas-OFFinder-predicted)	GAATTCTTAGGCGGATCCtagagcatatggggtttcca
PCSK9_W156X off target primer-IR site-2 (Cas-OFFinder-predicted)	ccctacacagtgtttttctc
PCSK9_W156X off target primer-OF site-3 (Cas-OFFinder-predicted)	gtggcttcctttctccaggg
PCSK9_W156X off target primer-OR site-3 (Cas-OFFinder-predicted)	gagcacagagactccatcgg
PCSK9_W156X off target primer-IF site-3 (Cas-OFFinder-predicted)	GAATTCACAGTGGGATCCgagcctgttctcctaggatg
PCSK9_W156X off target primer-IR site-3 (Cas-OFFinder-predicted)	ctgaaaacaccgccttacct
PCSK9_W156X off target primer-OF site-4 (Cas-OFFinder-predicted)	gcgcagggtagaaacattgc
PCSK9_W156X off target primer-OR site-4 (Cas-OFFinder-predicted)	gacctcctgagtgttcagcc
PCSK9_W156X off target primer-IF site-4 (Cas-OFFinder-predicted)	GAATTCGCCAATGGATCCgcctgggcaatagagtgaga
PCSK9_W156X off target primer-IR site-4 (Cas-OFFinder-predicted)	atgttggggaccagttacgc
PCSK9_W156X off target primer-OF site-5 (Cas-OFFinder-predicted)	ttgtaaggatcagtgttgcccac
PCSK9_W156X off target primer-OR site-5 (Cas-OFFinder-predicted)	ggacaagcctcgcagaatcag
PCSK9_W156X off target primer-IF site-5 (Cas-OFFinder-predicted)	GAATTCCAGATCGGATCCtcctccgcacagtgtcgggt
PCSK9_W156X off target primer-IR site-5 (Cas-OFFinder-predicted)	acccccgtgctggagcccat
PCSK9_W156X off target primer-OF site-6 (Cas-OFFinder-predicted)	cctgaactcccacttcaccc
PCSK9_W156X off target primer-OR site-6 (Cas-OFFinder-predicted)	agtgtgagatgctgtgaggc
PCSK9_W156X off target primer-IF site-6 (Cas-OFFinder-predicted)	GAATTCACTTGAGGATCCtgccctagtgctgagctcat
PCSK9_W156X off target primer-IR site-6 (Cas-OFFinder-predicted)	tcttgggtgaagctagggag
PCSK9_W156X off target primer-OF site-7 (Cas-OFFinder-predicted)	gcactgccaaacacatctcc
PCSK9_W156X off target primer-OR site-7 (Cas-OFFinder-predicted)	ggggcatcggtctctctcta
PCSK9_W156X off target primer-IF site-7 (Cas-OFFinder-predicted)	GAATTCGATCAGGGATCCgaatctgagtgggccttgga
PCSK9_W156X off target primer-IR site-7 (Cas-OFFinder-predicted)	ttgggcttccacatggcatg
PCSK9_W156X off target primer-OF site-8 (Cas-OFFinder-predicted)	agaggtctgggtctcaacca
PCSK9_W156X off target primer-OR site-8 (Cas-OFFinder-predicted)	atggattgcaggatgcctct
PCSK9_W156X off target primer-IF site-8 (Cas-OFFinder-predicted)	GAATTCTAGCTTGGATCCctacagagagtcattgtgac
PCSK9_W156X off target primer-IR site-8 (Cas-OFFinder-predicted)	ttcatttagcccatttccca
PCSK9_W156X off target primer-OF site-9 (Cas-OFFinder-predicted)	gctcgctcctgggaagattt
PCSK9_W156X off target primer-OR site-9 (Cas-OFFinder-predicted)	aagcctctgaagccctcaac
PCSK9_W156X off target primer-IF site-9 (Cas-OFFinder-predicted)	GAATTCGGCTACGGATCCtcacttaacagcctcccctt
PCSK9_W156X off target primer-IR site-9 (Cas-OFFinder-predicted)	tgggtgttccaagctagaga
PCSK9_W156X off target primer-OF site-10 (Cas-OFFinder-predicted)	ggcactggagcaactctctt
PCSK9_W156X off target primer-OR site-10 (Cas-OFFinder-predicted)	gtccttgctctgcctctcag
PCSK9_W156X off target primer-IF site-10 (Cas-OFFinder-predicted)	GAATTCAGTCAAGGATCCaacacagccactgtctccag
PCSK9_W156X off target primer-IF site-10 (Cas-OFFinder-predicted)	tcagcgtcttggttagcgcc
PCSK9_W156X off target primer-OF site-11 (Cas-OFFinder-predicted and PEM-seq-detected)	ggacaagatagcctcgcagc
PCSK9_W156X off target primer-OR site-11 (Cas-OFFinder-predicted and PEM-seq-detected)	cttgtattgtccctgcggttg
PCSK9_W156X off target primer-IF site-11 (Cas-OFFinder-predicted and PEM-seq-detected)	GAATTCCTTGTAGGATCCtctgcccttaatgacgttca
PCSK9_W156X off target primer-IR site-11 (Cas-OFFinder-predicted and PEM-seq-detected)	tgtgttttccgagctacgga
PCSK9_W156X off target primer-OF site-12 (Cas-OFFinder-predicted and PEM-seq-detected)	ggccatgatgaggccatagtc
PCSK9_W156X off target primer-OR site-12 (Cas-OFFinder-predicted and PEM-seq-detected)	agacattgtgcagattgaccca
PCSK9_W156X off target primer-IF site-12 (Cas-OFFinder-predicted and PEM-seq-detected)	GAATTCATCACGGGATCCtttgcctagtctccaggcaa
PCSK9_W156X off target primer-IR site-12 (Cas-OFFinder-predicted and PEM-seq-detected)	acaagacgagaggaatagga
PCSK9_W156X off target primer-OF site-13 (Cas-OFFinder-predicted and PEM-seq-detected)	tgggtgttcaggaggtaaagag
PCSK9_W156X off target primer-OR site-13 (Cas-OFFinder-predicted and PEM-seq-detected)	aatggcagtcattaggaacc
PCSK9_W156X off target primer-IF site-13 (Cas-OFFinder-predicted and PEM-seq-detected)	GAATTCGTAGAGGGATCCtggtctttgggagctccttg
PCSK9_W156X off target primer-IR site-13 (Cas-OFFinder-predicted and PEM-seq-detected)	atgggctcaggagccagtgt
PCSK9_W156X off target primer-OF site-14 (PEM-seq-detected)	ggttgtccctccaccctcta
PCSK9_W156X off target primer-OR site-14 (PEM-seq-detected)	agagggtcctctgggttcag
PCSK9_W156X off target primer-IF site-14 (PEM-seq-detected)	GAATTCATGTCAGGATCCctgattctgctgactctgac
PCSK9_W156X off target primer-IR site-14 (PEM-seq-detected)	tggggtctcctcagctccac
PCSK9_W156X off target primer-OF site-15 (PEM-seq-detected)	cgtggagaatccggtcagtg
PCSK9_W156X off target primer-OR site-15 (PEM-seq-detected)	ccgcctgctagaggaattgg
PCSK9_W156X off target primer-IF site-15 (PEM-seq-detected)	GAATTCCCGTCCGGATCCtagccaagccaccaatgctc
PCSK9_W156X off target primer-IR site-15 (PEM-seq-detected)	cagtccctcactatcaaacg
PCSK9_W156X off target primer-OF site-16 (PEM-seq-detected)	acacctcaaagagcagcctc
PCSK9_W156X off target primer-OR site-16 (PEM-seq-detected)	ctgttgccagtttgccatcc
PCSK9_W156X off target primer-IF site-16 (PEM-seq-detected)	GAATTCGTCCGCGGATCCgagcttctccttctcccctt
PCSK9_W156X off target primer-IR site-16 (PEM-seq-detected)	ccactgtgacacaggccttg
PCSK9_W156X off target primer-OF site-17 (PEM-seq-detected)	attccagctctgcccttgtc

PCSK9_W156X off target primer-OR site-17 (PEM-seq-detected)	ctgggtgtccttcagtggac
PCSK9_W156X off target primer-IF site-17 (PEM-seq-detected)	GAATTCGTGAAAGGATCCgccctggtcctcaagcctgt
PCSK9_W156X off target primer-IR site-17 (PEM-seq-detected)	tccatggggacagcggctat
APOE_R158C on target primer-OF	aggcctacaaatcggaactgg
APOE_R158C on target primer-OR	ctcgaaccagctcttgaggc
APOE_R158C on target primer-IF	GAATTCTATAATGGATCCatgctcggccagagcaccga
APOE_R158C on target primer-IR	acgcggccctgttccaccag
APOE_R158C off target primer-OF site-1 (Cas-OFFinder-predicted)	acccagtttagtgacggctg
APOE_R158C off target primer-OR site-1 (Cas-OFFinder-predicted)	cagagccttggctcctgaat
APOE_R158C off target primer-IF site-1 (Cas-OFFinder-predicted)	GAATTCCACGATGGATCCggctgtctgtttgcatcctg
APOE_R158C off target primer-IR site-1 (Cas-OFFinder-predicted)	gctactggttccctgaacaa
APOE_R158C off target primer-OF site-2 (Cas-OFFinder-predicted)	cgccaaaatctcaaccgtgaa
APOE_R158C off target primer-OR site-2 (Cas-OFFinder-predicted)	ctgcctctggtctgcattgg
APOE_R158C off target primer-IF site-2 (Cas-OFFinder-predicted)	GAATTCCACTCAGGATCCtgcagacagaagccaaaggg
APOE_R158C off target primer-IR site-2 (Cas-OFFinder-predicted)	aggctgaagtgaatggcgtc
APOE_R158C off target primer-OF site-3 (Cas-OFFinder-predicted)	cttggggacctgggatgaac
APOE_R158C off target primer-OR site-3 (Cas-OFFinder-predicted)	ggacacagatcctgatgggc
APOE_R158C off target primer-IF site-3 (Cas-OFFinder-predicted)	GAATTCCAGGCGGGATCCtaaatgctggctctgcctcc
APOE_R158C off target primer-IR site-3 (Cas-OFFinder-predicted)	agctgcttaaccccttcgtg
APOE_R158C off target primer-OF site-4 (Cas-OFFinder-predicted)	catttctgggtggctgaggt
APOE_R158C off target primer-OR site-4 (Cas-OFFinder-predicted)	tgctgccctggctaattcaa
APOE_R158C off target primer-IF site-4 (Cas-OFFinder-predicted)	GAATTCCATGGCGGATCCtgtgggaaatatactaaggc
APOE_R158C off target primer-IR site-4 (Cas-OFFinder-predicted)	tctgagcctcagtttcctca
APOE_R158C off target primer-OF site-5 (Cas-OFFinder-predicted)	tgaacttcacccagcacctg
APOE_R158C off target primer-OR site-5 (Cas-OFFinder-predicted)	cactgggtctctgtggtgag
APOE_R158C off target primer-IF site-5 (Cas-OFFinder-predicted)	GAATTCCATTTTGGATCCtctggtatttacccaggatt
APOE_R158C off target primer-IR site-5 (Cas-OFFinder-predicted)	tcttggtgccctggcagaca
APOE_R158C off target primer-OF site-6 (Cas-OFFinder-predicted)	agcccagggaatatggatgc
APOE_R158C off target primer-OR site-6 (Cas-OFFinder-predicted)	cctgagtcggaaaccactgt
APOE_R158C off target primer-IF site-6 (Cas-OFFinder-predicted)	GAATTCCCAACAGGATCCacgtctgagcccagcttcct
APOE_R158C off target primer-IR site-6 (Cas-OFFinder-predicted)	cagctggccatatttacagg
APOE_R158C off target primer-OF site-7 (Cas-OFFinder-predicted)	ctccaagctgaacaggaccc
APOE_R158C off target primer-OR site-7 (Cas-OFFinder-predicted)	gagacacagagaagcgggac
APOE_R158C off target primer-IF site-7 (Cas-OFFinder-predicted)	GAATTCCGGAATGGATCCaagaagctaatggggtgggc
APOE_R158C off target primer-IR site-7 (Cas-OFFinder-predicted)	ctcctctctgtaggcccagt
APOE_R158C off target primer-OF site-8 (Cas-OFFinder-predicted)	gccggaatagagcactcgtt
APOE_R158C off target primer-OR site-8 (Cas-OFFinder-predicted)	ctctccgaagctgttcaccc
APOE_R158C off target primer-IF site-8 (Cas-OFFinder-predicted)	GAATTCCTAGCTGGATCCgtgcattatgagcactcaga
APOE_R158C off target primer-IR site-8 (Cas-OFFinder-predicted)	ctgtccactagaatgcaagc
APOE_R158C off target primer-OF site-9 (Cas-OFFinder-predicted)	tcttcccatggcaggcaatc
APOE_R158C off target primer-OR site-9 (Cas-OFFinder-predicted)	cctcccctttcatcctgctc
APOE_R158C off target primer-IF site-9 (Cas-OFFinder-predicted)	GAATTCCTATACGGATCCcacctcccgtctgtcagtct
APOE_R158C off target primer-IR site-9 (Cas-OFFinder-predicted)	atagtgtgaaaccccaccgc
APOE_R158C off target primer-OF site-10 (Cas-OFFinder-predicted)	gctgcccactcactcctaag
APOE_R158C off target primer-OR site-10 (Cas-OFFinder-predicted)	agcagctgtgtgtttcccat
APOE_R158C off target primer-IF site-10 (Cas-OFFinder-predicted)	GAATTCCTCAGAGGATCCgcagcaagcacatacctcct
APOE_R158C off target primer-IR site-10 (Cas-OFFinder-predicted)	atgctttgcaggggttttgg
APOE_R158C off target primer-OF site-11 (Cas-OFFinder-predicted and PEM-seq-detected)	aaagagtaaattcagtccttcctca
APOE_R158C off target primer-OR site-11 (Cas-OFFinder-predicted and PEM-seq-detected)	taattgaaagctgctgcctcaga
APOE_R158C off target primer-IF site-11 (Cas-OFFinder-predicted and PEM-seq-detected)	GAATTCGACGACGGATCCtcctgactgtgcctcatatg
APOE_R158C off target primer-IR site-11 (Cas-OFFinder-predicted and PEM-seq-detected)	aagcactgactacctggccc
APOE_R158C off target primer-OF site-12 (Cas-OFFinder-predicted and PEM-seq-detected)	tagaaagcaggcatacaag
APOE_R158C off target primer-OR site-12 (Cas-OFFinder-predicted and PEM-seq-detected)	gagaagggcagatgaaaa
APOE_R158C off target primer-IF site-12 (Cas-OFFinder-predicted and PEM-seq-detected)	GAATTCTAATCGGGATCCaaagcaggcatacaagtacc
APOE_R158C off target primer-IR site-12 (Cas-OFFinder-predicted and PEM-seq-detected)	cagagtgagacctgtggaag
